# A comparative genomic analysis of targets of Hox protein Ultrabithorax amongst distant insect species

**DOI:** 10.1038/srep27885

**Published:** 2016-06-14

**Authors:** Naveen Prasad, Shreeharsha Tarikere, Dhanashree Khanale, Farhat Habib, L. S. Shashidhara

**Affiliations:** 1 Indian Institute of Science Education and Research Pune, 411008, India

## Abstract

In the fruitfly *Drosophila melanogaster*, the differential development of wing and haltere is dependent on the function of the Hox protein Ultrabithorax (Ubx). Here we compare Ubx-mediated regulation of wing patterning genes between the honeybee, *Apis mellifera*, the silkmoth, *Bombyx mori* and *Drosophila*. Orthologues of Ubx are expressed in the third thoracic segment of *Apis* and *Bombyx*, although they make functional hindwings. When over-expressed in transgenic *Drosophila*, Ubx derived from *Apis* or *Bombyx* could suppress wing development, suggesting evolutionary changes at the level of co-factors and/or targets of Ubx. To gain further insights into such events, we identified direct targets of Ubx from *Apis* and *Bombyx* by ChIP-seq and compared them with those of *Drosophila*. While majority of the putative targets of Ubx are species-specific, a considerable number of wing-patterning genes are retained, over the past 300 millions years, as targets in all the three species. Interestingly, many of these are differentially expressed only between wing and haltere in *Drosophila* but not between forewing and hindwing in *Apis* or *Bombyx*. Detailed bioinformatics and experimental validation of enhancer sequences suggest that, perhaps along with other factors, changes in the cis-regulatory sequences of earlier targets contribute to diversity in Ubx function.

Transcription factors (TFs) of Hox family govern segment specific identity across the phyla by regulating diverse developmental pathways. The functional significance of Hox family proteins in shaping morphological traits is well documented. However it remains to be determined as to how the intrinsic (i.e. alterations in the orthologous protein sequence) and extrinsic (i.e. additional cofactors, changes in activity etc.) factors are coordinated to ultimately orchestrate evolution of morphological features. Here we address this question in the context of evolution of wing number and morphology in insects. Insects are the first to evolve flight appendages. Most insects have four wings (all directly contribute to the flight), while beetles and flies have only one pair of wings. In beetles (such as *Tribolium castenum*), forewing is modified into thick protective organ called elytra. In dipterans, such as the fruitfly *Drosophila melanogaster*, the hindwing is modified into haltere, which functions as a balancing organ. Except in few early insect groups, all four-winged insects also display differences in the forewing and hindwing morphology (such as size, shape and decorative patterns).

In *Drosophila*, the differential development of wing and haltere is dependent on the function of Ultrabithorax (Ubx) in haltere primordia^1^, a member of Hox family of TFs. Intriguingly, it has been shown that *Tribolium* Ubx functions to suppress elytra development and to specify wing identity in T3^2^. In the lepidopteran insect, *Precis coenia* (or *Junonia coenia*), Ubx is required to generate differences in the eyespots between fore- and hindwings^3^. Thus, Ubx likely functions to diversify T2 and T3 appendages in all insect species. This makes Ubx function central to studies on the evolution of wing number and morphology amongst the diverse insect groups.

Although there are significant differences in Ubx sequences between *Drosophila* and crustacean Arthropods^4^,^5^, ectopic-expression of Ubx derived from a non-winged arthropod, such as Onychophora, is sufficient to induce wing-to-haltere transformations in *Drosophila*^6^. Assuming that this is true amongst diverse insect groups, it is likely that Ubx may have acquired new targets and/or new cofactors in *Drosophila* lineage to repress wing development and specify haltere development. Bioinformatics and experimental validation of all these possibilities needs identification of direct targets of Ubx from diverse insect species. In this study we have employed comparative genomics to postulate the mechanisms used by functionally similar Ubx to favor the development of morphologically different organs in T3 segment. Hox proteins bind to ‘TAAT’ core sequence, although the precise mechanism by which Hox genes recognize and regulate their targets is poorly understood. We have, therefore, undertaken experimental identification of targets of Ubx from other insect species, to complement the bioinformatics based analysis of different genomes. In earlier studies, others and we have reported identification of putative direct targets of Ubx in the haltere discs of *Drosophila*^7^,^8^,^9^. In this study, we identified putative direct targets of Ubx by ChIP-seq from two different insect species, which have been diverging from *Drosophila* for nearly 300 million years. We chose the honeybee, *Apis mellifera* (http://hymenopteragenome.org/beebase/), a member of Hymenoptera and the Silkmoth, *Bombyx mori* (http://sgp.dna.affrc.go.jp/index.html and http://silkworm.genomics.org.cn/), a member of Lepidoptera, both of which represent two major divergences that resulted in separation of Dipterans from the other Endopterygotes (Fig. 1A)^10^. Unlike *Drosophila*, they both have two pairs of wings: one each on second and third thoracic segments. Their genome sequences have also been reported^11^,^12^,^13^. We compared the putative targets of Ubx in *Apis* and *Bombyx* to the previously reported lists of targets in *Drosophila*^7^,^8^ to understand the events downstream of Ubx as focus for molecular evolution leading to haltere specification in dipterans. While only about 15–20% of the putative targets of Ubx in *Apis* and *Bombyx* were common to those in *Drosophila*, a large proportion of these common targets are known to function during wing development in *Drosophila*. Interestingly, several of these common targets are differently expressed between wing and haltere in *Drosophila*, while the same targets are not differentially regulated between fore- and hindwings in *Apis* and *Bombyx*. Thus, rather than binding of Ubx per se, these dissimilarities are likely an outcome of differences in the binding of other TFs near Ubx-binding regions. Ubx protein may collaborate with such cofactors to regulate the expression of target genes. To assess this idea, we identified and experimentally tested one of the enhancers of *vestigial (vg*) from *Apis*, which is equivalent to the quadrant enhancer of vg in *Drosophila*. When expressed in transgenic *Drosophila*, unlike its fly counterpart, the *vg* enhancer from *Apis* is expressed in both wing and haltere discs. Thus, our observations suggest that, changes in the modes of regulation of earlier targets are one of the factors that may contribute to the evolution of Ubx function.

## Results and Discussion

### Expression pattern of Ubx in *Apis* is different from that of *Bombyx* and *Drosophila*

**Figure 1:**
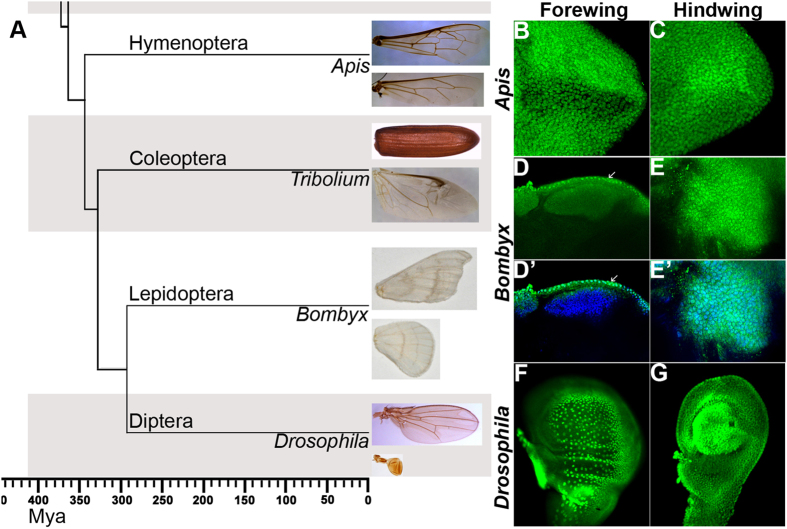
(**A**) Phylogenetic relationship of four major types of endopterygote insects and their wing morphology. The dendrogram was drawn based on the relationship reported in Zdobnov and Bork (2007)^39^. This traces the divergence of 4 major orders of endopterygote insects for nearly 350 million years. Hymenopterans diverged first, then Coleopterans followed by separation of Lepidoptera and Diptera. They are represented by *Apis, Tribolium, Bombyx* and *Drosophila*, respectively. Forewing and hindwing morphology of each insect is shown in the panel on the right side. Note, while *Apis* and *Bombyx* show near identical fore- and hindwings, in *Tribolium*, the forewing is modified into a thick protective organ called elytra. In *Drosophila*, the hindwing is modified into small club shaped organ called haltere. (**B–G**) Expression patterns of Ubx in developing fore- and hindwing in *Apis* and *Bombyx*. (**B,C**) Forewing (**B**) and hindwing (**C**) buds of 5^th^ instar *Apis* larvae stained with anti-Ubx_*Apis*_ antibodies. Note, Ubx is expressed in both the wingbuds, while levels are marginally higher in the hindwing buds. (**D,E**) Forewing (**D**) and hindwing (**E**) buds of 4^th^ instar *Bombyx* larvae stained with anti-Ubx_*Bombyx*_ antibodies. While Ubx is expressed in the hindwing proper, its expression in the forewing bud is restricted to only the peripodial membrane (arrows). D’ and E’ show the same discs as in (**D**) and (**E**), respectively showing both DAPI (blue) and anti-Ubx_*Bombyx*_ (green) staining. (**F,G**) Wing (**F**) and haltere (**G**) discs of 3^rd^ instar *Drosophila* larvae stained with anti-Ubx_*Drosophila*_ antibodies. Ubx is strongly expressed in haltere cells, while its expression in the wing disc is restricted to the peripodial membrane. In (**B–E**), proximal end is shown towards the left and the distal end towards the right. In (**F,G**), anterior is towards left and ventral up.

All the Hox proteins as well as several non-Hox TFs share highly conserved homeodomains, while N-terminal sequences show much divergence (Suppl. Fig. 1A). To avoid cross-reactivity due to such homologous sequences between different proteins, we raised polyclonal antibodies against N-terminal regions specific to *Apis* Ubx and *Bombyx* Ubx for our study. The antibodies against Ubx from *Apis* or *Bombyx* did not cross-react with Ubx from *Drosophila* (Suppl. Fig. 1B–F). Using these antibodies, we first determined the patterns of Ubx expression in fore- and hindwing buds of *Apis* and *Bombyx*. We observed that Ubx is expressed in both forewing and hindwing buds in *Apis*, although expression is stronger in hindwing buds (Fig. 1B,C). In *Bombyx* forewing buds, Ubx expression is restricted to the peripodial membrane, whereas it is strongly expressed in developing hindwing buds (Fig. 1D,E). In *Drosophila* too, Ubx is expressed only in the haltere discs (Fig. 1F,G). While it is expressed in the peripodial membrane of wing discs, it has no role in wing development. Earlier reports suggest that in butterflies too Ubx expression is restricted to hindwing buds, although they have not reported it expression in the peripodial membrane for the fore- or hind-wing buds^13^,^14^. We did not detect any peripodial membrane in *Apis*. As the expression pattern of Ubx in *Bombyx* is similar to its expression pattern in *Drosophila*, it is likely that restriction of Ubx expression to T3 and subsequent posterior segments may have evolved after the divergence of hymenopterans (such as *Apis*) from other endopterygotes (in the coleopteran *Tribolium* too, Ubx is expressed only in the third thoracic segment^2^). The characteristic expression pattern of Ubx across the three systems further validated our choice to analyze and compare the transcriptional targets of Ubx.

### Ubx from *Apis, Bombyx* and *Tribolium* can suppress wing development in *Drosophila*

**Figure 2:**
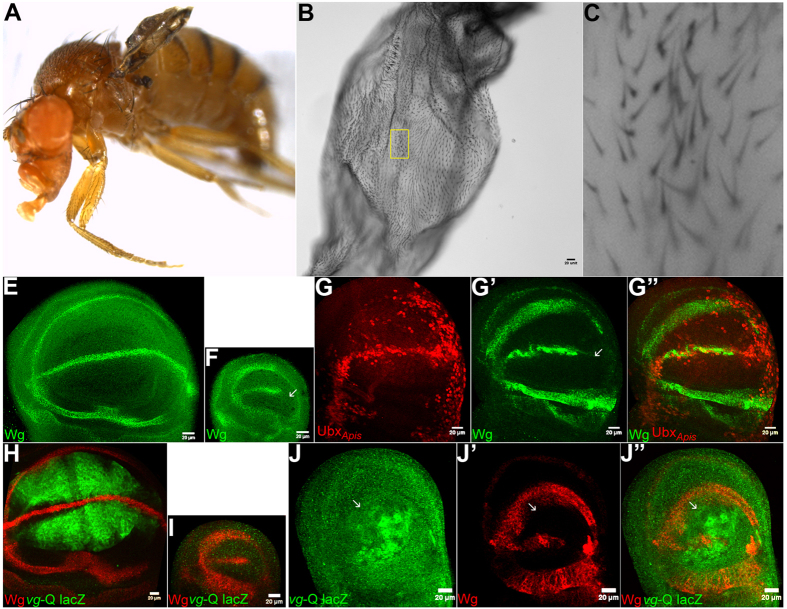
Wing to haltere transformations in *Drosophila* caused by the ectopic-expression of Ubx_*Apis*_. (**A–C**) *vg*-GAL4/UAS-Ubx_*Apis*_ adult fly with reduced and deformed wing. The separated wing is shown at higher magnifications (**B,C**). Note trichomes are shorter and more densely arranged. (**E,F**) Wildtype larval wing (**E**) and haltere (**F**) discs stained for Wg (green). While Wg is expressed in in both anterior and posterior compartments in the wing disc, it is not expressed in the posterior compartment (arrow in **F**) of the haltere disc. (**G**) *vg*-GAL4/UAS-Ubx_*Apis*_ larval wing disc stained for Wg (green) and Ubx_*Apis*_ (red). Note repression of Wg by Ubx_*Apis*_ is only in the posterior compartment (arrow in G’), an indication of homeotic transformation. (**H,I**) *vg* quadrant-lacZ larval wing (**H**) and haltere (**I**) discs stained for lacZ (green). (**J**) *vg*-GAL4/UAS-Ubx_*Apis*_; *vg* quadrant-lacZ larval wing disc stained for lacZ (green) and Wg (red). Note, non cell-autonomous repression of *vg* quadrant-lacZ. Repression of Wg in the posterior compartment is more pronounced in J (arrows) than in G. In all imaginal discs, posterior is to the right and ventral to the top.

A comparison of amino acid sequence of Ubx from *Apis*, *Bombyx*, *Tribolium* and *Drosophila* is shown in Suppl. Fig. 1G. In addition to the respective homeodomains, several other regions are conserved amongst these four species. By contrast, most of the differences are concentrated within the N-terminal regions alone. To assess whether the Ubx proteins between different species are functionally conserved we undertook ‘*in vivo*’ trans-heterologous assays. To this end, we generated transgenic *Drosophila* strains that enabled ectopic-expression of Ubx derived from *Apis* and *Bombyx* and tested the ability of respective transgenes to suppress wing development and induce wing-to-haltere transformations. As no information is available regarding the function of Ubx during wing development in *Apis* or *Bombyx*, as a positive control we also included Ubx from *Tribolium*, whose function has been genetically examined in a similar assay. Interestingly, the *Tribolium* homolog of Ubx is involved in conferring the wing identity to the third thoracic segment^2^. When over-expressed in *Drosophila* however, *Tribolium* Ubx behaved similarly as *Drosophila* Ubx, although this was examined only at the level of suppression of leg development^4^. We observed that ectopic-expression of Ubx from all three insect species caused suppression of wing development, to a similar degree caused by the ectopic-expression of *Drosophila* Ubx (Fig. 2; Suppl. Fig. 2). With *Apis* Ubx, we also observed change in trichome size and/or organization that resembled wing-to-haltere transformations (Fig. 2C). For more precise analysis of effects of ectopic-expression of different Ubx proteins, we monitored the expression patterns of Wingless (Wg) and Vestigial (Vg), the two well-characterized targets of Ubx. Wg is expressed only in the anterior compartment of haltere discs, while it is expressed in both anterior and posterior compartments of wing discs. For Vg, we used its quadrant enhancer reporter transgene strain, which is expressed in wing discs, but not in haltere discs^14^,^15^. We observed wing-type to haltere-type transformation in the expression of patterns of Wg for Ubx of all the four insect species (Fig. 2G; Suppl. Fig. 2). They also caused moderate to strong suppression of Vg expression in wing discs (Fig. 2J; Suppl. Fig. 2). As the levels of expression from different transgene are not the same, we couldn’t ascertain if there is any difference in the function of Ubx from different species. Nevertheless, the fact that ectopic expression of Ubx from all the four species cause repression of Wg expression only in the posterior compartment of wing discs supported earlier prediction that Ubx protein itself has not evolved amongst these diverse insect groups^6^.

### Ubx targets similar developmental functions in *Apis, Bombyx* and *Drosophila*

The ability of Ubx from *Apis*, *Bombyx* and *Tribolium* to suppress *Drosophila* wing development in the transgenic assay suggests that evolution of halteres in dipterans could be ascribed to changes in the events downstream or parallel of Ubx. To test this idea, we identified putative direct targets of Ubx in the progenitors (wing buds) of both fore- and hindwing of *Apis* and *Bombyx* by ChIP-seq analyses. We used a strain of *Apis* being maintained in the laboratory of M Beye at Dusseldorf, Germany. We used larvae from a single hive seeded by a single-male inseminated queen to reduce the variations in ChIP-seq data. Genomic sequence of this strain is identical to the published sequence. For *Bombyx*, several pure-breed/isogenic strains are available, which are being used for commercial purposes. We identified the one, Daizo, which had least deviations from the published genome (of the race Daizo50T).

**Figure 3:**
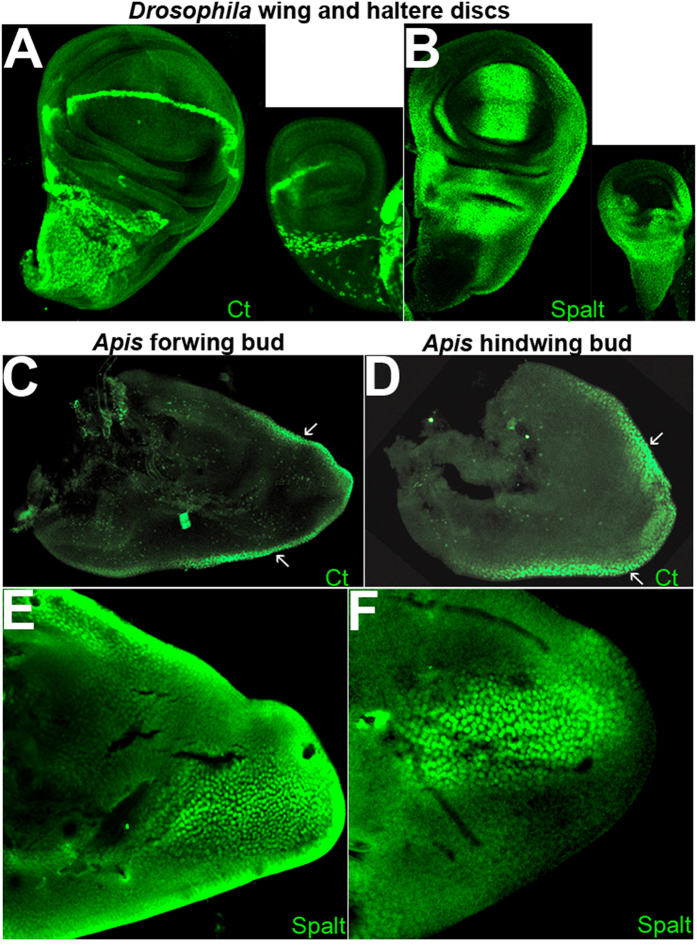
Unlike in *Drosophila* wing discs, many wing-patterning genes are not differentially expressed in *Apis* wing buds. (**A,B**) *Drosophila* wing and haltere discs stained for Ct (**A**) and Spalt (**B**). Note Ct is not expressed in the posterior compartment of the haltere discs, while Spalt is absent from the entire pouch of the haltere. Outside the pouch, the expression of Spalt is comparable between wing and haltere discs. (**C–F**) Forewing (**C,E**) and hindwing (**D,F**) buds of Apis stained for Ct (**C,D**) and Spalt (**E,F**) using antibodies against their *Drosophila* homologues. Their expression patterns in *Apis* are similar to the patterns seen in *Drosophila* wing discs. However, there is no difference between the forewing and hindwing buds in *Apis* unlike wing and haltere in *Drosophila*. Arrows in (**C**) and (**D)** indicate D/V boundary.

Most studies related to patterning events during *Drosophila* wing development use wandering third instar larval wing to ascertain expression patterns of various genetic factors. This is mainly due to the fact that this developmental stage marks the end of patterning events and beginning of morphogenesis. Identification of putative direct targets of Ubx in *Drosophila* was also performed using wandering third instar larval wing and haltere imaginal discs^7^,^8^,^9^. We therefore, first, determined equivalent stage in *Apis* and *Bombyx* by studying the expression patterns of key wing patterning genes. An earlier report suggests that expression patterns of several wing-patterning genes in hymenopteran wing buds are similar to those in *Drosophila* wing discs^16^. We determined the expression patterns in *Apis* wing buds of Cut (Ct), Spalt (Sal), Extradenticle (Exd) and Engrailed (En) by RNA *in situ* and/or by immuno-histochemistry using antibodies against the *Drosophila* proteins. They all showed similar expression patterns in *Apis* wing buds as in *Drosophila* wing discs (Fig. 3A–F; Suppl. Fig. 3A–G). Based on the expression patterns of these genes, in particular that of Spalt and Ct (which are targets of A/P and D/V signaling centres), we identified early fifth instar larval stage in *Apis* as equivalent to wandering third instar larval stages in *Drosophila*. In the case of *Bombyx*, expression patterns reported earlier for Wg, Nubbin and Dll^17^, suggest that 4^th^ instar larval stage is equivalent to wandering third instar larval stages in *Drosophila*.

For both *Apis* and *Bombyx*, we carried out ChIP-seq (in 2 replicates each) on chromatin extracted from both fore- and hindwing buds using polyclonal antibodies against the N-terminal fragment of corresponding Ubx (see Suppl. Text and Suppl. Figs 4 and 5 for details). Genes nearest to the immunoprecipitated chromatin fragments were identified as putative targets of Ubx see Suppl. Text for details). For Ubx in *Apis*, large numbers of genes were identified as putative targets in both forewing (583) and hindwing (1396) buds (Suppl. Fig. 5A). As expected, since Ubx is expressed in both the fore- and hindwing buds, a very large number of them were common to both the tissues (305; Suppl. Fig. 5C). The larger number of hindwing-specific peaks observed in *Apis* could be possibly attributed to the differences in the levels of Ubx expression between the two wing buds. In *Bombyx*, wherein Ubx is expressed only in the developing hindwing, we identified 871 genes as putative targets of Ubx in hindwing buds as opposed to 245 genes in the forewing buds (Suppl. Fig. 5B). Only a small fraction of these (43 genes) was overlapping between the two wing buds (Suppl. Fig. 5D). The forewing-specific peaks of Ubx in *Bombyx* could be due to its expression in the peripodial membrane (and not the forewing proper). For comparison of function of Ubx between the three species, we only considered those putative targets that have *Drosophila* orthologues. Of 1396 putative targets in *Apis* hindwing buds, 1182 have orthologues in *Drosophila*. Amongst 871 putative targets of Ubx in the *Bombyx* hindwing buds, 548 have orthologues in *Drosophila* (Suppl. Fig. 5A,B).

Choo *et al*.^8^ have reported 1147 genes as putative targets of Ubx in *Drosophila*, identified by ChIP-chip analysis of haltere discs expressing Ubx::YFP transgene. Agrawal *et al*.^7^ have reported 570 genes as putative targets of Ubx in *Drosophila*, identified by ChIP-chip analysis of Cbx wing discs using anti-Ubx antibodies specific to the N-terminal region of the protein. 219 of these targets were common to those reported by Choo *et al*.^8^ (Suppl. Fig. 6A). In yet another study, Slattery *et al*.^9^ have reported 3400 genes as putative targets of Ubx identified ChIP-chip analysis using antibodies against the full-length protein. As in this study, there is a possibility that non-Ubx homeodomain-containing proteins may have also been pulled down, we only used datasets reported by Agrawal *et al*.^7^ and Choo *et al*.^8^ for all comparative analyses.

**Figure 4:**
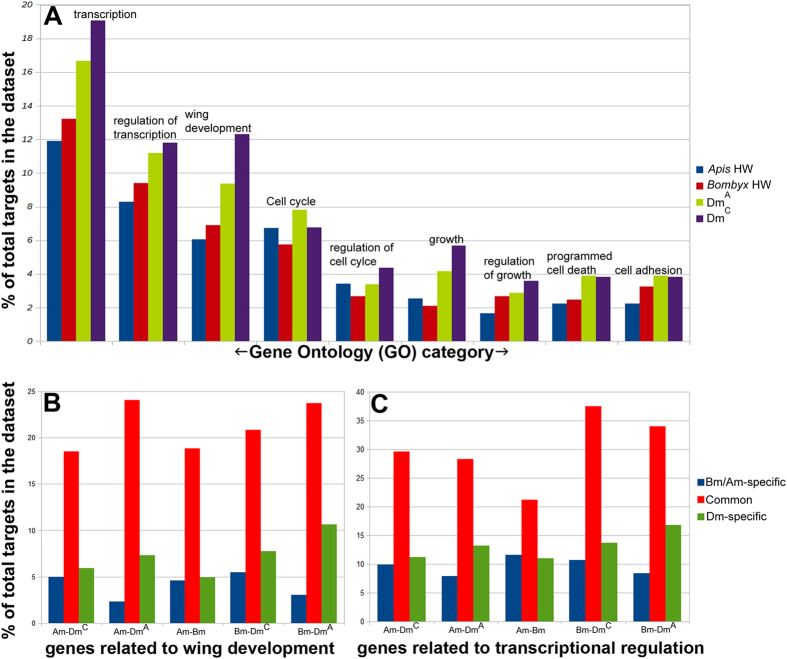
Targets of Ubx in *Drosophila*, *Apis* and *Bombyx* belong to similar functional groups. Bar diagrams here show frequency distribution of various ontological groups in different datasets as labeled. (**A**) Shows gene ontological distribution amongst all targets of Ubx. Note, gene ontological distribution of targets of Ubx is similar across all the species. However, there is an increase in the number of targets within a functional group in *Drosophila* than in *Bombyx* than in *Apis*. Gene ontological groups analysed are as follows (numbers in parenthesis are GO category number): transcription (GO-06350); regulation of transcription (GO-45449); wing development (GO-35220); cell cycle (GO-07049); regulation of cell cycle (GO-51726); growth (GO-40007); regulation of growth (GO-40008); programmed cell death (GO-12501) and cell adhesion (GO-07155). (**B,C**) show proportion of genes implicated in wing development (**B**) or transcriptional regulation (**C**) amongst the targets that are either specific to a given species or common between two species. The proportion of genes implicated in wing development or transcriptional regulation is much higher amongst the genes that are common between any two species compared to species-specific targets. Gene ontology is based on the information available for *Drosophila* genes.

Not only that many putative targets are common to all the three species (three-way comparison between *Apis*, *Bombyx* and two studies of *Drosophila*) (Suppl. Fig. 6), several important regulators of wing development in *Drosophila*, such as En, Hh, Vg and Legless, are amongst them (Suppl. Table 1). However, owing to different methods of identifying targets of Ubx in *Drosophila*^7^,^8^ and in *Apis*/*Bombyx* reported here, these comparisons might not be comprehensive. We therefore, investigated if there is a common pattern (or species-specific differences) in the functional groups of genes that are identified as putative targets of Ubx. We examined the distribution of various gene ontological groups amongst the putative targets of Ubx (using DAVID^18^). The distribution of targets across various ontological groups and their relative proportions (for example, how many targets amongst the total are cell-cycle regulators or cell-adhesion proteins) were very similar across all the three species suggesting that no new functional group has evolved as a target of Ubx in any lineage including the Diptera (Fig. 4A).

We then examined relative distribution of ontological groups between targets that are specific to a given species and that are common between two species. Main purpose was to identify those functional groups that are specifically enriched in targets that are specific to *Drosophila* and/or amongst targets that are common to *Drosophila* and *Apis*/*Bombyx*. In all the three pairwise comparisons of putative targets of Ubx (*Apis*-*Bombyx*; *Apis*-*Drosophila* and *Bombyx*-*Drosophila*), targets that are common to the two species had several ontological groups over-represented than in species-specific targets (Fig. 4B,C; Suppl. Fig. 7A–E). It is particularly interesting and relevant that genes implicated in such specialized function as wing development are over-represented by more than 4-fold amongst the targets that are common between *Apis/Bombyx* and *Drosophila* when compared to *Drosophila*-specific targets (Fig. 4C). This suggests their positive selection during evolution as targets of Ubx rather than any other non-causal associations.

### Common targets of Ubx are modulated in different ways in different insects

**Figure 5:**
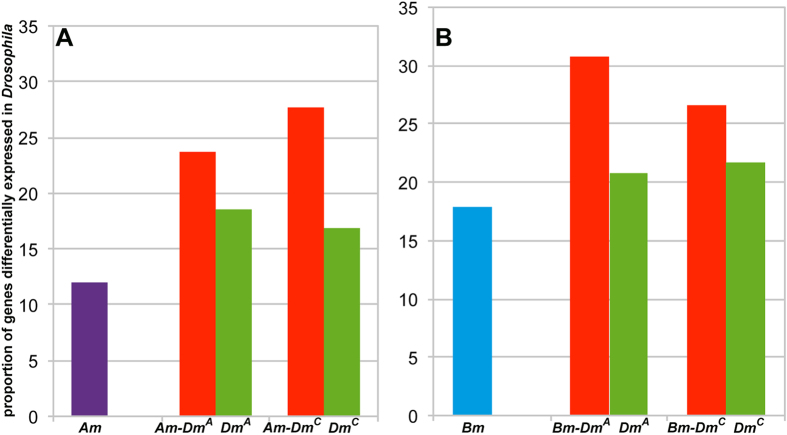
Comparison of targets of Ubx in different species to the genes that are differentially expressed between wing and haltere in *Drosophila*. Fly genes that are considered here as differentially regulated between wing and haltere were pooled from microarray analyses reported by Mohit *et al*.^19^ and Pavlapoulos *et al*. (2007)^20^. They were compared to the targets of Ubx in *Apis* (**A**) and *Bombyx* (**B**) determined in this study by ChIP-seq. Comparisons were also done for those genes that were either specific to a given species or common between two species (as indicated on the images). Marginally higher proportions of targets of Ubx that are common to *Drosophila* and *Apis* or *Bombyx* are differentially expressed between wing and haltere discs in *Drosophila*.

Although it is remarkable that several important wing-development-related genes are common targets of Ubx for nearly 300 million years, what is more relevant is the effect of binding of Ubx on their regulation. It is important to assess the functional relevance as mere binding or physical association does not ensure transcriptional activity. We thus investigated if there are differences in their expression patterns. First, we compared putative targets of Ubx that are common between *Apis* and *Drosophila*, between *Bombyx* and *Drosophila* and *Drosophila*-specific targets with the list of differentially expressed genes between wing and haltere determined by genome-wide microarray studies^19^,^20^. We found that a higher proportion of common targets are represented in the microarray data compared to the *Drosophila*-specific targets (Fig. 5A,B). This suggests that these common targets are indeed relevant to Ubx function, at least, in *Drosophila*.

Next, we examined the expression patterns in *Apis* and *Bombyx* of few selected orthologues of those genes of *Drosophila*, which are differentially regulated between wing and haltere. We noticed that genes such as Spalt, Cut and Extradenticle (we used antibodies against their *Drosophila* orthologues) are not differentially expressed between developing forewing and hindwing buds in *Apis* (Fig. 3C–F; Suppl. Fig. 3B). This was also true for *vg*, *Distal-less* and *wg* as detected by RNA *in situ* on *Apis* wing buds (Suppl. Fig. 3D–F). This supports earlier reports on identical expression patterns between fore- and hindwing buds of Wg in the ant (hymenopteran^16^) and Wg and Dll in *Bombyx*^17^,^21^. Furthermore, preliminary transcriptome analysis for fore- and hindwing buds in *Bombyx* suggested near identical patterns (Suppl. Fig. 8A). Only 241 genes showed a minimum of 2 fold difference in their expression levels between fore- and the hindwing buds. Of this only 10 are common to putative direct targets of Ubx in the hindwing as determined by ChIP-seq (Suppl. Fig. 8B). This suggests that mere binding of Ubx (as determined by ChIP analysis here) is not an indication of their regulation. Earlier reports suggest that while binding of *Drosophila* Ubx to its targets is instructive, it is not necessarily sufficient to confer regulation^7^. It is, therefore, possible that Ubx has acquired ability to regulate expression of certain genes that are important for wing development only in the dipteran lineage. As Ubx protein itself has not evolved to acquire this ability and it actually binds to these targets *in vivo* in four-winged species too, a most likely possibility is differences in enhancer sequences of those targets.

### Comparison of putative regulatory sequences of targets of Ubx across the three species

In the preceding sections we have shown that genes implicated in wing development in *Drosophila* are specifically enriched amongst the putative targets of Ubx that are common to *Drosophila* and *Apis* or *Bombyx* (Fig. 4B,C). Moreover, relatively higher proportion of these are differentially expressed in *Drosophila* (Fig. 5A,B), while many of these are not differentially expressed in *Apis* and *Bombyx*. Taken together these observations suggest that evolutionary changes that have resulted in the differential regulation of these genes in dipterans could be one of the major factors in the evolution of diversity in Ubx function. It is possible that evolutionary changes that have accrued in these genes over 300 million years may have made them more responsive to Ubx regulation.

Several kinds of changes in cis- and trans-factors may drive evolution of gene expression patterns. These include promoters, enhancers, insulator elements, transcriptional cofactors, miRNA, long non-coding RNA etc. We examined the putative cis-regulatory sequences to identify changes near Ubx-binding regions that may throw light on possible evolutionary changes that have made these genes more sensitive to Ubx binding. While changes could have happened anywhere in non-coding regions, we chose to map binding sites for TFs around Ubx-binding sites (determined by ChIP-seq). The rationale for this was, one: for our understanding of haltere evolution, regulation by Ubx is more important than stand-alone regulation by any other TF. Two: identification of true enhancer sequences (which evolve at a faster rate due to low selection pressure) across species is difficult. In this context, experimentally determined (by ChIP) Ubx-binding sites serve as reference points to compare equivalent regions.

**Figure 6:**
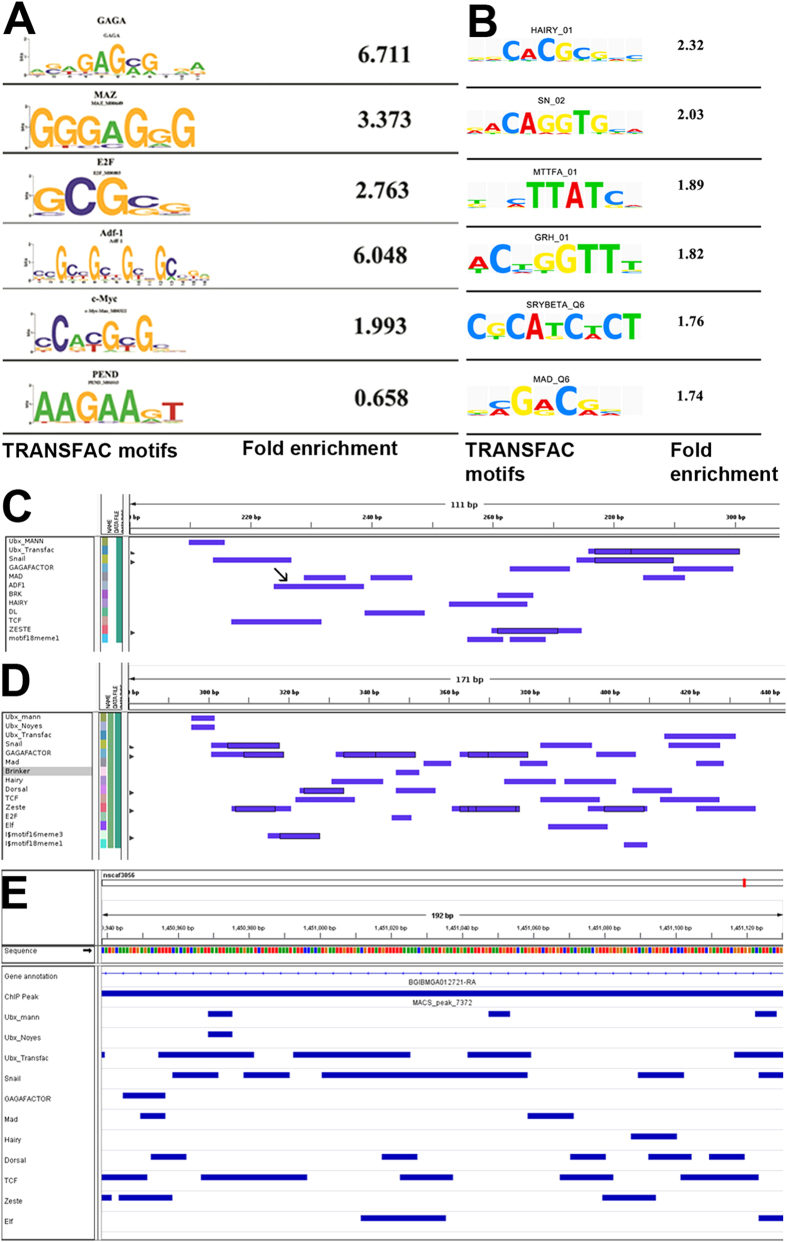
Bioinformatics analyses of putative enhancer sequences of targets of Ubx in *Apis* and *Bombyx*. (**A,B**) Enrichment of binding sites for various TFs in the sequences pulled down by anti-Ubx_*Apis*_ from *Apis* (**A**) and by anti-Ubx_*Bombyx*_ from *Bombyx* (**B**) hindwing buds identified using TRANSFAC. Motifs are based on information available for TFs available in *Drosophila*. These are similar to the ones reported for putative targets of Ubx in *Drosophila*^7^. In both (**A**) and (**B**), numbers indicate fold differences in the frequency of occurrence of a given motif between sequences pulled down by ChIP and non-coding sequences randomly collated from *Apis* or *Bombyx* genome. Similar enrichment for binding sites for GAF, Adf-1, E2F etc. is reported for *Drosophila*^7^. (**C–E**) TRANSFAC analysis of quadrant enhancer of *vg* in *Drosophila* (**C**) to its equivalent region in *vg* gene of *Apis* (**D**) and *Bombyx* (**E**). Note a similar array of TFs bind around Ubx binding sites in all the three species. However, Adf-1 binding site is absence in *Apis* and *Bombyx*.

We first carried out macro-analysis of all ChIP-pulled down sequences using MEME^22^ and Weedersuite^23^ for common motifs across all species. Earlier work suggested that in *Drosophila*, the Ubx binding sequence – ATAATG/C – is not specifically enriched in ChIP-pulled down sequences when compared to the rest of the non-coding part of the genome. Instead, sequences that are recognized by other TFs such as GAF, ADF-1, TCF, MAD, etc. were found significantly enhanced^7^. This and additional experimental evidence suggested that Ubx functions by identifying and interacting with certain TF complexes regulating gene expression during wing development^7^. We observed similar scenario for the putative targets of Ubx in both *Apis* and *Bombyx*. ChIP-pulled down sequences did not show enrichment for Ubx-binding motifs, instead, they showed significant enrichment for motifs to which various other TFs (such as GAF, ADF-1, c-MYC, MAZ etc.) bind and regulate gene expression during development (Fig. 6A,B; Suppl. Fig. 9A–C). Both Ubx binding motifs and other motifs (thought to be recognition sequences of other TFs) were, however, based on studies in *Drosophila* and the information was extrapolated to *Apis* and *Bombyx* genomes. We next carried out similar analysis amongst different groups of targets (*Drosophila*-specific targets, common to *Drosophila* and *Apis* (or *Bombyx*) or specific to *Apis* (or *Bombyx*) targets. The list of potential co-factors or co-TFs and the frequency of occurrence of their binding sites around Ubx-binding sites were similar, although not identical between the three species (Suppl. Fig. 9D,E). Overall, we did not find any clue in these analyses on the nature of differences in the sequences around the regions bound by Ubx in *Apis, Bombyx* and *Drosophila*. While these observations may explain retention of certain genes as targets by Ubx in different insect lineages, it fails to explain acquisition of large number of new targets in a specific lineage. This requires further detailed investigation of non-coding sequences, which may include comparison amongst several members within a lineage.

### Enhancer of *vestigial* from *Apis* is resistant to Ubx regulation unlike its counterpart from *Drosophila*

Next, we specifically focused on why certain common targets are differentially expressed in *Drosophila*, but not in *Apis* or *Bombyx*. Enhancers of certain *Drosophila* genes such as *Dll, Spalt, vg*, *CG13222* etc. are well studied in the context of regulation of their expression during wing development as well as their regulation by Ubx during haltere specification. As identifying equivalent enhancers from other distant species is difficult, we decided to employ following three criteria

1. The enhancer sequence should be conserved amongst the closely related species.
2. The enhancer sequences are embedded within introns and thus are readily searchable
3. enhancer sequences are relatively less spread out, preferably a one contiguous short fragment regulating gene expression, albeit partially.

Based on these three criteria, we short-listed *vg* for such comparative analysis. In *Drosophila*, *vg* has two enhancers, the D/V boundary enhancer (present within the 2^nd^ intron^24^) and the non-DV boundary quadrant enhancer (present with in the 4^th^ intron^25^). Both are intronic and are well characterized. They drive reporter gene expression in transgenic experiments in a pattern identical to that of the endogenous protein. Importantly, while the expression pattern of the D/V enhancer is identical between wing and haltere, the quadrant enhancer of *vg* is expressed only in the wing disc and Ubx suppresses its expression in the haltere disc (Weatherbee *et al*., 1998; Shashidhara *et al*., 1999). We decided to directly compare the ability of enhancer/s from *Apis* to regulate gene expression during wing and haltere development in a transgenic context in *Drosophila*. At the time of this analysis the *Bombyx* genome assembly was incomplete and not many closely related genomes are yet to be sequenced, so we decided to carry out this analysis only for *Apis*.

**Figure 7:**
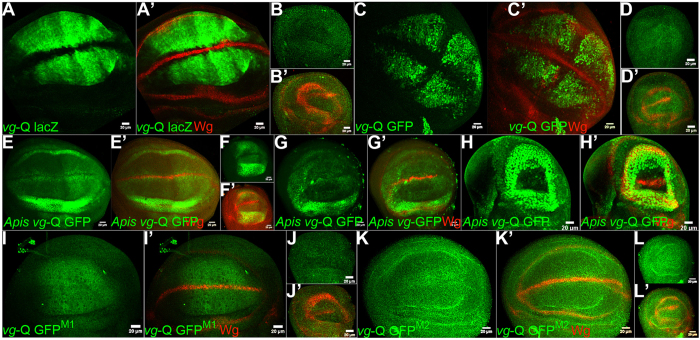
The putative quadrant enhancer of *vg* of *Apis* drives the report gene GFP in transgenic *Drosophila* in a pattern similar to the quadrant enhancer of *vg* of *Drosophila*. (**A,B**) *Drosophila vg*-Q lacZ wing (**A**) and haltere (**B**) discs stained for LacZ (green) and Wg (red). Wg marks the D/V boundary. *Drosophila vg*-Q lacZ is expressed in non-D/V cells of the wing pouch, but is completely absent from the haltere discs. This transgenic line is reported by Kim *et al*. (1996). (**C,D**) *Drosophila vg*-Q GFP wing (**C**) and haltere (**D**) discs stained for GFP (green) and Wg (red). Note, the expression pattern is very similar to *vg*-Q lacZ. (**E,F**) *Apis vg*-Q GFP wing (**E**) and haltere (**F**) discs stained for GFP (green) and Wg (red). Note strong GFP expression in both wing and haltere discs. In both the discs, expression is limited to non-D/V cells of the pouch and in the presumptive hinge. Expression along the A/P boundary is lower, suggestive of quadrant expression pattern similar to the *Drosophila vg*-Q GFP. (**G,H**) *vg*-GAL4/ *Apis vg*-Q GFP; UAS-Ubx_*Drosophila*_ (**G**) and *vg*-GAL4/ *Apis vg*-Q GFP; UAS-Ubx_*Apis*_ (**H**) wing discs stained for GFP (green) and Wg (red). Note there is no change in the expression pattern of *Apis vg*-Q GFP. This is contrary to the severe repression observed for *Drosophila vg*-Q lacZ (Fig. 3J). (**I,J**) None of the two different mutant versions of *Apis vg*-Q GFP show any GFP staining in wing or haltere discs suggesting that mutating Adf-1 binding sites to MAD-binding sites may have resulted in complete loss of its activation during wing development. GFP that is seen in (**I**) is not nuclear and appears to be non-specific. In *Apis vg*-Q GFP^M1^, Adf-1 binding site tggctgccgtcgcgat is replaced with MAD1-binding site (as in the *Drosophila* genome) gctgcccgccgc. In *Apis vg*-Q GFP^M2^, Adf-1 binding site tggctgccgtcgcgat was replaced with MAD1-binding site (as in the *Apis* genome) gccgtcgc.

We identified a putative quadrant enhancer within the 4^th^ intron (see Suppl. Text for details) of *vg* gene of *Apis*, which is highly conserved amongst hymenopteran species, whose genome sequences are available (Suppl. Fig. 10). This harbors Ubx-binding sites and was detected as a positive peak in the ChIP-seq for Ubx targets. We generated transgenic flies driving the expression of GFP using this enhancer (hereafter referred to as *Apis vg*-Q GFP). While such transgenics are reported earlier for the quadrant enhancer of *Drosophila vg* (Kim *et al*., 1996; Fig. 7A,B), for a better comparison we also generated new GFP-tagged enhancer transgenic strains (hereafter referred to as *vg*-Q GFP). Interestingly, although the two species have diverged for nearly 300 million years, *Apis vg*-Q GFP and *vg*-Q GFP showed comparable expression patterns in *Drosophila* wing discs (Fig. 7C,E). Similar to *vg*-Q GFP, *Apis vg*-Q GFP is not expressed in the D/V and A/P boundary cells. This confirms that the intronic DNA fragment tested here is likely a true enhancer of *vg* in *Apis* and is regulated during wing development in a pattern similar to its counterpart in *Drosophila*. However, while *vg*-Q GFP was completely repressed in haltere discs, *Apis vg*-Q was found expressed in both wing and haltere discs, at similar levels and patterns (Fig. 7D,F). *Apis vg*-Q was also resistant to ectopic expression of Ubx (both *Drosophila* and *Apis* Ubx; Fig. 7G,H) in wing discs unlike its *Drosophila* counterpart^14^,^15^. These observations confirm functional differences in the enhancers of *vg* between *Drosophila* and *Apis*. It is likely that *Drosophila* variant may have evolved an additional feature(s) enabling Ubx-mediated repression through this enhancer.

### Enhancers of some common targets show subtle differences in the array of binding sites for transcription factors

To get deeper insight into the sequence elements in *Drosophila vg*-Q that are responsible for its repression by Ubx, we carried out detailed comparison of enhancer sequences from both the species at single nucleotide resolution. We observed that *vg*-Q has very similar array of TF binding sites around Ubx-binding regions in both *Apis* and *Drosophila* (Fig. 6C,D). In *Drosophila vg*-Q, there is a motif, ‘TGGCTGCCGTCGCGAT’, which is predicted as recognition sequence for Adf-1. This motif is conspicuously absent in *Apis vg*-Q. GCCGTCGC, a motif present within the putative Adf-1 binding sequence, also serves as recognition sequence for MAD1. Interestingly, MAD1-binding sites are present in both *Drosophila vg*-Q (Fig. 6C) and *Apis vg*-Q (Fig. 6D). While Ubx-MAD1 interaction has been already demonstrated biochemically^26^, it remains to be tested whether Ubx also interacts with Adf-1. Interestingly, both Ubx^7^ and Adf-1^28^ have been shown to interact with GAF. It is, therefore, possible that presence of Adf-1 binding site/s is the reason for the ability of Ubx to repress *vg* expression in *Drosophila*. To test if presence of Adf-1-binding site in *Drosophila vg*-Q is critical for its repression in haltere discs, we generated transgenic flies in which these sequences were altered. Rationale was to see if this makes them resistant to Ubx and thus show similar patterns of expression between wing and haltere discs.

As *vg* expression in *Drosophila* is dependent on the binding of MAD1 to its quadrant enhancer^27^, we wanted to ensure that MAD1-binding sites remain intact. We generated two transgenic flies. *Drosophila vg*-Q^M1^: Adf-1-binding motif (TGGCTGCCGTCGCGAT) was replaced by MAD1-binding motif found in *Apis* (GCTGCCCGCCGC). *Drosophila vg*-Q^M2^: Adf-1-binding motif (TGGCTGCCGTCGCGAT) was replaced by MAD1-binding motif found in *Drosophila* (GCCGTCGC). These transgenic flies, however, did not show any specific expression pattern of GFP in wing disc itself (neither in haltere discs; Fig. 7I–L). Perhaps those binding sites are required to activate *vg* expression in the *Drosophila* wing pouch. Ubx may repress its expression in the haltere disc by interfering with this MAD/Adf-1 complex. If this is true, then the question arises what activates *vg* in the *Apis* wing discs in the absence of Adf-1 binding sites and how *Apis vg*-Q is able to drive GFP expression in *Drosophila* wing discs. Nevertheless, taken together these data suggest that acquisition of binding sites for additional TFs without altering binding *per se* of Ubx could be a major driving force to generate more diversity in Ubx function.

We also compared enhancer sequences of few other genes. *wg, ct* and *spalt* are differentially regulated between wing and haltere in *Drosophila*, while no detectable differences in their expression patterns were observed between fore- and hindwings in *Apis* (Fig. 3C–F and Suppl. Fig. 3D,F). We compared sequences around Ubx-bound region (as determined by ChIP-seq) near or within these genes. We observed that in spite of divergence for 300 million years, *Apis* and *Drosophila* have very similar array of TF binding sites around Ubx-binding regions (Suppl. Fig. 11). However, we also noticed, a few differences between the two species. For example, in the gene *ct*, *Drosophila* has an En-binding site near the Ubx-binding site, which is absent in *Apis* (Suppl. Fig. 11). Consistent with this, *ct* is repressed only in the posterior compartment of wildtype haltere (Fig. 3A) and is not differentially expressed between fore- and hindwing in *Apis* (Fig. 3C,D). We also compared non-coding sequences around Ubx-binding regions of *vg* of *Drosophila* and *Bombyx*. The putative quadrant enhancer of *vg* of *Bombyx* (which is on 4^th^ intron and is a positive in ChIP-seq analysis) harbors similar array of binding sites for various TFs as in *Drosophila*. In *Bombyx* too, however, the Adf-1 binding sites are absent (Suppl. Fig. 11). We observed similar trends when non-coding sequences around Ubx-binding regions of *E*(*Spl*)*mβ*, *brinker (brk*) and *hedgehog (hh*) were compared between *Drosophila* and *Bombyx* (Suppl. Fig. 11).

In conclusion, comparative genomic analysis reported here suggests that two related yet distinct factors determine the course of evolution of morphological characteristics regulated by Ubx. One, acquisition of new targets. Large number of species-specific targets suggest that in each lineage Ubx may have a somewhat unique function. Second, molecular alterations in the mode of regulation of existing targets. This is supported by the observations that certain targets of Ubx are not differentially expressed between fore- and hindwings in *Apis* and *Bombyx*, while they are differentially expressed between wing and haltere in *Drosophila*. It is possible that both these attributes together may function as major drivers of the evolution of wing morphology in T3 downstream of Ubx. Detailed analyses of similarities and differences in the enhancers that drive *vg* expression in *Apis* and *Drosophila* suggest that evolutionary changes in the enhancer sequences that allow for novel modes of regulation by Ubx may help canalization of Ubx function in the context of diversification of dorsal appendages from T2-type (forewing) to T3-type (haltere) morphology.

## Experimental Methods

### *Drosophila*-related work

#### Fly stocks were obtained from various sources

GAL4 drivers used in this study are *vg*-GAL4^29^ and Ubx-GAL4^30^. UAS lines used are UAS-Ubx_*Drosophila*_^31^, UAS-Ubx_*Apis*_, UAS-Ubx_*Bombyx*_ and UAS-Ubx_*Tribolium*_ (all three generated in this study). Other fly strains that were used are *Drosophila* quadrant *vg*-lacZ^25^, *Drosophila vg*-Q GFP, *vg*-Q GFP^M1^, *vg*-Q GFP^M2^ and *Apis vg*-Q GFP, all four generated in this study).

#### Generation of Transgenic flies

cDNA for Ubx from *Apis* and *Bombyx* were isolated by RT-PCR, sequence-verified and cloned into pUAST-FLAG vector. Full length cDNA for Ubx from *Tribolium* was from SB Carroll, which was further cloned into pUAST-FLAG vector. Enhancers of *Apis vg* and *Drosophila vg* were isolated by genomic PCR, sequence-verified and cloned into pH Stinger vector. The mutant versions of *vg* enhancer of *Drosophila* was generated by PCR using appropriate primers, sequence-verified and cloned into pH Stinger vector. Details of all primers used in this study are provided in the Suppl. Text. Transgenic flies for all constructs were generated by Fly-injection facility at C-CAMP, Bangalore, India.

#### Histology

Immunochemical staining on imaginal discs were performed as described earlier^32^. Antibodies used were rabbit anti-GFP (1:3000; Invitrogen); chicken anti-GFP (1:500; Invitrogen); mouse anti-β-galactosidase (1:500; 40-1a, DSHB); mouse anti-Ubx^33^; mouse anti-Wingless^34^ (1:200) and mouse anti-Cut^35^ (1:10). Monoclonal antibodies against Wingless (Wg), Cut (Ct) and β-galactosidase (βgal) were obtained from DSHB, Iowa, USA. The secondary antibodies conjugated with different fluorophores were obtained from Invitrogen. Fluorescent images were taken using Zeis LSM 710 or Zeiss LSM 780 confocal microscope. Adult wings were processed for imaging as described in Shashidhara *et al*. (1999)^15^.

### *Apis*-and *Bombyx*-related work (see Suppl. Text for additional details)

#### Generation of polyclonal antibody against Ubx of Apis mellifera

cDNAs corresponding to the N- terminal region of Ubx of *Apis* and *Bombyx* (excluding the conserved homeodomain and YPWM motif) were generated cloned into pET15b vector using standard methods. See Suppl. Text for additional details on cloning, expression and purification of the protein and raising of antibodies. The specificity of antibodies was confirmed by immuno-histochemistry on *Apis* (or *Bombyx*) wing buds and by Western Blot hybridization on purified proteins as well as wing bud lysates (Suppl. Fig. 1D–F). They did not cross-react to *Drosophila* Ubx (either the purified protein or the haltere disc lysate).

#### Histology

The method was essentially same as Patel *et al*. (1989)^32^ with certain modifications as described in the Suppl. Text. Following antibodies against *Drosophila* proteins were directly used on *Apis* tissues. Monoclonal anti-Cut^35^ (1:5) 2B10, anti-Extradenticle^36^ (1:5) EXD B11M, anti-Engrailed^32^ (1:5) 4D9 were obtained from DSHB. Anti-Spalt^37^ (1:50) was a kind gift from Tiffany Cook, CCHMC.

Anti-Ubx (*Apis*) antibodies, generated in this study, were used at a dilution of 1: 3000 for antibody staining on *Apis* wingbuds and at a dilution of 1:6000 for Western blot hybridization. Anti-Ubx (*Bombyx*) antibodies, generated in this study, were used at a dilution of 1: 500 for antibody staining on *Bombyx* wingbuds and at a dilution of 1:2500 for Western blot hybridization.

#### Chromatin immune-precipitation (ChIP) and Transcriptome analyses

We used a modified protocol that was used earlier for *Drosophila* wing discs^7^,^38^. All ChIP-Seq and RNA-Seq experiments (described below) and data analyses conform to The Functional Genomics Data Society (http://fged.org/) guidelines. Detailed description is provided in the Suppl. Text.

Raw data is available on http://www.ncbi.nlm.nih.gov/geo/query/acc.cgi?acc=GSE71847 (for data generated by ChIP-Seq using anti-*Apis* Ubx antibodies), http://www.ncbi.nlm.nih.gov/geo/query/acc.cgi?acc=GSE71990 (ChIP-Seq using anti-*Bombyx* Ubx antibodies) and http://www.ncbi.nlm.nih.gov/geo/query/acc.cgi?acc= GSE71988 (RAN-seq for total RNA isolated from fore- and hindwing buds of *Bombyx* larvae).

## Additional Information

**How to cite this article**: Prasad, N. *et al*. A comparative genomic analysis of targets of Hox protein Ultrabithorax amongst distant insect species. *Sci. Rep.* **6**, 27885; doi: 10.1038/srep27885 (2016).

## Supplementary Material

Supplementary Information
