# Supplementary material for: A comparative genomic analysis of targets of Hox protein Ultrabithorax amongst distant insect species
Source: Sci Rep. 2016 Jun 14;6:27885. doi: 10.1038/srep27885 (PMC4906271; doi:10.1038/srep27885)
Supplement: Supplementary Information [file srep27885-s1.doc]

**A comparative genomic analysis of targets of Hox protein Ultrabithorax amongst distant insect species**

Naveen Prasad^1#^, Shreeharsha Tarikere^1$^, Dhanashree Khanale, Farhat Habib, LS Shashidhara*

Indian Institute of Science Education and Research Pune, 411008, India.

*Correspondence to [ls.shashidhara@iiserpune.ac.in](mailto:ls.shashidhara@iiserpune.ac.in)

1: co-first authors

#: current address: Centre for Neural Circuits and Behaviour, The University of Oxford, Tinsley Building, Mansfield Road, Oxford OX1 3SR, UK

$: current address: Department of Organismic and Evolutionary Biology, Harvard University, Cambridge, Massachusetts, USA

**SUPPORTING INFORMATION**

**Experimental Methods**

**P-element constructs for the generation of transgenic flies**

All constructs were sequence-verified before generating the transgenic flies.

1. Primers for amplifying Ubx from total cDNA of *Apis* larvae by RT-PCR

Forward Primer: 5’ TCTCCGTCCTCTCGACAGAT 3’

Reverse Primer: 5’ GGAGGAGGGAAGGATTGAAG 3’

(Submitted to Genbank Accession no. GU169707)

The amplicon was cloned into pGEM-T easy vector. Ubx from this vector was amplified using the following primers and subcloned into pUAST- flag vector.

Forward Primer: 5’ ACGG GGTACC ATGAACTCGTATTTTGAGCAGACTGC 3’

Reverse Primer: 5’ AGGC TCTAGA CTAGTTGGCCCCCTCCGG 3’

2. Primers for amplifying Ubx from total cDNA of *Bombyx* embryos by RT-PCR:

Forward Primer: 5’ ACGG GGTACC ATGAACTCTTACTTCGAGCAGGGTG 3’

Reverse Primer: 5’ ATGC TCTAGA TTAATGTTCGGGGTGTCCCTGG 3’

The amplicon was directly subcloned into pUAST vector.

3. Ubx from *Tribolium*:

The cDNA (in pGEX vector) for ‘a’ isoform of *Tribolium* Ubx was obtained by S Carroll. The insert was amplified using following primers for subcloning into pUAST vector.

Forward Primer: 5’ ACGG GAATTC TATGAACTCTTACTTCGAGCAGAGC 3'

Reverse Primer: 5' AGGC TCTAGA CTAATTCGGGTCCACTTGTGCG 3'

4. *vg* enhancer from *Apis* (*Apis vg-*Q GFP)

As the region identified as putative enhancer of *vg* is AT rich, following primers were used to amplify a larger fragment using genomic DNA as template.

Forward primer: 5’ TTCGTCGCTCGAATTCACCA 3’

Reverse primer: 5’ TTACGGCGGTTTGCTTTTGG 3’

The amplicon was subcloned into pGEM T easy vector (Promega). This construct was used as template to amplify the putative enhancer of *vg* using following primers.

Forward primer: 5’ GCTCTAGA CTTCTCGCGAGAAACGAGAGGC 3’

Reverse primer: 5’ CGGGATCC GTGGACAGTGACGAGGACACG 3’

The amplicon was further sub-cloned into pH stinger vector.

5. *vg* enhancer from *Drosophila* (*Drosophila vg*-Q GFP)

Forward primer: 5’ TGCTCTAGAGGAGCTCCCTCCGGAG 3’

Reverse primer: 5’ CGCGGATCCATCGATTGTACTTTGTCGTTTCTAATTG 3’

The amplicon was first sub-cloned into pGEM T easy vector (Promega) then transferred to pH stinger vector using restriction enzymes Xba1 and BamH1.

6. Generation of mutant versions of *Drosophila* quadrant *vg* enhancer

We used a PCR based approach. Briefly, *vg* enhancer in the pEM T easy vector was used as a template and amplification was carried out using primers that had mutations at their 3’ end (as shown below). The amplicon was ligated and used for the transformation of DH5α. The positive plasmids were sequenced to confirm the mutation. Finally the mutated fragment in pGEMT easy vector was subcloned into pH stinger vector using restriction enzymes Xba1 and BamH1.

Mutant form of *vg* enhancer from *Drosophila* (*vg*-Q GFP^M1^: wherein Adf-1 binding site tggctgccgtcgcgat is replaced with gctgcccgccgc)

Forward primer: 5’ CGCCGCTCGACAACTTTGGCCGGC 3’

Reverse primer: 5’ GGCAGCAGCACAAACATTACACATCCCC 3’

Mutant form of *vg* enhancer from *Drosophila* (*vg*-Q GFP^M2^: (wherein Adf-1 binding site tggctgccgtcgcgat was replaced with gccgtcgc)

Forward primer: 5’ TCGCTCGACAACTTTGGCCGGC 3’

Reverse primer: 5’ CGGCAGCACAAACATTACACATCCCC 3’

***Apis*-related work**

Initial characterization of wing buds of honeybees and antibody staining were carried out on *Apis mellifera* collected from various beekeepers in India. Subsequently honeybee hives of *Apis mellifera* were maintained at IISER, Pune for post-ChIP follow up studies. The bees were fed with sugar solution and pollen substitute. For each replicate of chromatin immune-precipitation (ChIP) studies, larvae were collected from a single hive seeded by a single queen artificially inseminated using semen from a single drone. These bee larvae were collected at Prof. Martin Beye's lab at Heinrich Heine University, Dusseldorf.

*1. Identification of wing buds and histology*

Comparing their shape across different hymenopteran insects, wing buds in honeybee larvae were identified. Their dorsal location in the second and third thoracic segments was also an identification criterion.

The immune-histochemcial method was essentially same as Patel et al. (1989)^1^ with minor modifications. Fixation was with 4% formaldehyde for 25 minutes at room temperature. Both primary and secondary antibodies were incubated with 0.5% BSA.

*2. Generation of polyclonal antibody against Ubx of Apis mellifera*

DNA fragment corresponding to the N- terminal region of Ubx of *Apis* (excluding the conserved homeodomain and YPWM motif) was generated using RT-PCR from total cDNA of honey bee larvae and cloned into pET15b vector using standard methods. Following primers with recognition sites for Nde1 and BamH1 were used to amplify desired region of Ubx.

Forward Primer: 5’GGG AAT TCC ATA TGT ATT TTG AGC AGA CTG CG 3’

Reverse Primer: 5’CGC GGA TCC GTT GTT GCC AGG ACT CGA 3’

The clone was used to express the recombinant Ubx containing protein in BL21 DE3 strain of bacteria (expression was induced using 1mM IPTG). The expressed protein was purified using Qiagen Ni-NTA matrix according to manufacturer's guidelines. The purified protein (Suppl. Fig. 1B) was used to raise antibodies in rabbit according to standard 90-day protocol for antibody generation. Protein A column (Millipore) was used to purify the anti-Ubx and pre-immune sera. The IgG fractions were concentrated using Amicon Ultra 15 centrifugal device with 30000 NMWL. The concentrated antibodies were stored by stabilising them in 50% glycerol at -70 ^o^C. The specificity of antibodies was confirmed by immuno-histochemistry on *Apis* wing buds and by Western Blot hybridization on purified proteins as well as wing bud lysates (Suppl. Fig. 1D). They did not cross-react to *Drosophila* Ubx (either the purified protein or the haltere disc lysate).

*3. Chromatin immune-precipitation (ChIP):*

We used a modified protocol that was used earlier for *Drosophila* wing discs^2,3^.

*Preparation of chromatin*

Dissected larval heads with exposed wing buds were fixed in 1.5% formaldehyde (freshly prepared) for 30 minutes at room temperature with gentle rocking. The reaction was stopped by adding one tenth the volume of 1.375M glycine in PBS and shaking it for another 10 minutes. The larval heads were washed three times (10 minutes each) in ice cold PBS to completely wash off formaldehyde. The wing buds were dissected out in cold PBS, pooled in a microfuge tube with 300 µl of swelling buffer (normally 10 times the volume of wingbuds). For each experimental replicate, 100 fore wing buds and 120 hind wing buds were used. They were incubated on ice for 15 minutes and were dounced intermittently to rupture the cell membrane. The ruptured cells were spun at 2000 rpm for 5 min at 4 ºC and the pellet was re-suspended in 300 µl of sonication buffer. The mix was dounced twice and then sonicated in a Diagenode water bath with 30 s ON followed by 30s OFF cycle for 10 minutes at HIGH power. This was the ideal setting to obtain chromatin fragments of size ~500bp (Suppl. Fig. 4A). The sample was centrifuged at 14000 rpm for 15 minutes at 4 ^o^C. Supernatant was removed and flash frozen in liquid nitrogen and subsequently stored in -80 ^o^C.

*Immuno-precipitation*

Equilibrated protein A Sepharose was washed 3 times with sonication buffer. Sepharose was blocked for four hours in sonication buffer containing 1 mg/mL BSA. Chromatin, (earlier stored in -80 ^o^C) was thawed on ice and divided into 100µl aliquots for immune precipitation. The final volume of each aliquot was made to 1 mL using dilution buffer. BSA was added to a final concentration of 1 mg/ mL. The chromatin was pre-cleared by incubating it with protein A sepharose for two hours at 4 ^o^C with gentle shaking. Antibodies (anti-Ubx IgG or pre immune IgG; 5 µg) was added to the respective chromatin aliquots and rotated for 2 hours at 4 ^o^C. 40 µl of protein A sepharose (50% slurry) was then added and incubated overnight with constant rotation at 4 ^o^C. The mix was centrifuged at 3800 g for 5 minutes at 4 ^o^C. Supernatant was removed and the sepharose beads were washed two times with 1 mL sonication buffer, 1 mL wash buffer A, 1 mL wash buffer B and 1mL TE. 100µl elution buffer was added to each of the aliquots and placed on a vortex mixer for 10 minutes. The tube was centrifuged and the supernatant was removed and stored. Another round of elution was carried out with 75 µl of elution buffer. Both the fractions were mixed and saved.

*Reverse Cross linking and isolation of DNA*

The volume of eluate from each aliquot was made to 400 µl. 21µl of 4 M sodium chloride was added to the eluate and incubated at 65 ^o^C for 5 hours. This was incubated with 1 µl of RNaseA (10mg/mL) at 37 ^o^C for 1 hour. 4 µl of 0.5 M EDTA and 2µl of proteinase K (10 mg/mL) was then added and incubated at 42 ^o^C for 2 hours. DNA was extracted using phenol: chloroform: iso amyl (25:24:1) followed by washing using chloroform: iso-amyl alcohol (24:1). DNA was then purified by ethanol purification by adding 1 µl of glycogen (stock 20mg/ml) and 1/20th the volume of 4M sodium chloride (to a final concentration of 0.2 M) and 2.5 volumes cold ethanol. DNA was allowed to precipitate overnight at – 20 ^o^C. It was centrifuged, washed and re suspended in 10mM Tris, pH 8.

Sequencing: The ChIP fragments were sequenced by a commercial service provider Genotypic Technology Pvt. Ltd., Bangalore using Illumina platform using standard protocols provided by the manufacturer.

Raw data is available on <http://www.ncbi.nlm.nih.gov/geo/query/acc.cgi?acc=GSE71847>.

*4. Data analysis and different bioinformatics tools*

The ChIP-seq reads obtained as above were first checked for quality statistics using FastQC program. Model-based analysis of ChIP-Seq (MACS)^4^ version 1.4 was used to identify peaks from the aligned sequences. DNA isolated directly from the chromatin used for ChIP in each replicate was used as input DNA control. This DNA sample too was sequenced and was used to determine baseline for peak calling.

The peaks both conditions (i.e. IP using anti Ubx IgG and control i.e. IP using pre immune IgG) were identified against input DNA sequence (Suppl. Fig. 4C). The peaks were filtered with stringent criteria to help reduce false positives. (A) All the pre-immune IgG peaks were subtracted from the anti-Ubx IgG peaks. (B) Only those peaks were considered which had an FDR <= 1% and a fold enrichment greater than 10 over input (Suppl. Fig. 5A). (C) The peaks those were common to both the replicates after this filtering was used for subsequent applications.

Bedtools was used for various operations on genomic intervals. Genes were assigned to different peaks using standard bioinformatics approach. Briefly, if a gene was identified within 2 kb on either sides of the peak, or if the peak itself was found in a gene; the gene was considered as a putative Ubx target. Even if multiple genes were found within 2kb of the peak on either side, all of them were considered positive hits. All the peaks that didn’t have any genes within 2kb were searched for genes on either side and the first gene within 5kb on any side was taken as a positive hit. BioMart was used to identify the orthologs of these honeybee genes in *Drosophila* melanogaster. Venny was used as an online bioinformatics tool to determine the common and specific elements between datasets. DAVID was used as a tool to classify the genes based on their biological process and pathways with which they are associated. Motifs in the chipped DNA sequences of *Apis* were identified using MEME and Weeder. Only significant motifs (motifs with e value <= 0.01 in MEME and the most significant motifs reported by Weeder) were chosen for subsequent analysis. STAMP was used to align the motifs and representative motifs of each family was reported and used for further analysis. BedTools was used to generate 300 files of random DNA fragments of *Apis* genome, each containing 2400 fragments of length 1000bp each. MATCH program was used under 'minimise false positive' condition to determine the frequency at which the motifs occurred in the random as well as in chipped DNA sequences. Fold enrichment of motifs was calculated by dividing the occurrence of motifs in the chipped DNA fragment over that in random DNA sequences.

*5. Identification of putative quadrant enhancer of* vg *of* Apis

In *Drosophila*, *vg* has two enhancers, a D/V boundary enhancer present on the 2^nd^ intron and a non-DV boundary quadrant enhancer, which is present on the 4^th^ intron. As the non-DV quadrant enhancer in *Drosophila* shows a differential expression between wing and haltere and is regulated by Ubx, we wanted to identify a comparable enhancer in *Apis*. Using comparative approach, GB14312 was identified as the ortholog in *Apis* of *vg* (FBgn0003975) gene of *Drosophila*. Based on our ChIP-Seq data on *Apis*, we identified two Ubx binding sites in introns of GB14312*.* One on the 2^nd^ intron and the other on the 4^th^ intron. The coordinates of these Ubx bound regions are:

gnl|Amel_2.0|Group12.16 1358386 1358695

gnl|Amel_2.0|Group12.16 1354425 1355183

The 550-bp region around the Ubx-binding site in the 4th intron of *vg* in *Apis* is highly conserved across all the hymenopterans. We hypothesized this to be equivalent of *vg* quadrant enhancer of *Drosophila* (which is also on 4^th^ intron) and chose this region for further experimental validation.

***Bombyx*-related work:**

Silkworms maintained in Centre for Sericulture Research and Training Institute (CSR&TI) at Mysore, India at 25ºC were used for this study. Initially two races (Daizo and C108) were obtained from Central Sericultural Gemplasm Resources Centre (CSGRC), Hosur, India. They were reared on Mulberry (*Morus alba*) leaves as feed. Storing of silkworm eggs, hatching and staging of larvae were as per standard procedures used in silkworm labs world-wide.

Selected intronic and exonic sequences of both the races, Daizo and C108, were compared to the *Bombyx* genome sequence available in the public databases. *Bombyx* homologues of *Drosophila* *Cubitus interrruptus* and Cytoplasmic Actin A4 are well described and their exon, intron, CDS etc are well marked. They were amplified from Daizo and C108 and their sequences were compared to the published genome sequences. While both the races were found to be equally identical to the genome, we used the multivoltine race Daizo (derived from Daizo 50T, whole genome sequence of which has been determined) for all future experiments.

*1. Larval dissection and isolation of wing buds and histology*

The method to isolate larval wing buds in *Bombyx* and their location in the larval body was derived from studies on segmental transplantation of wing buds in fifth instar larvae^5^. Therefore, initially wing bud isolations were practiced on fifth instar larvae. The dissection was performed by making an incision in the center of the segment 2 for forewing and segment 3 for the hindwing. The wingbuds are attached to the bodywall with the help of a trachea that passes from anterior to posterior. The wingbuds were released by cutting the tracheae with minimal amount of it remaining in the bud. We observed that by the 5^th^ instar stage the wing venations are already well developed and perhaps not appropriate for studying the function of Ubx in pattern formation. The late fourth instar of the *Bombyx* larva was found to be equivalent to the 3^rd^ instar larval stages of *Drosophila* based on the expression patterns of key patterning genes^6^. We also observed that at this stage the wingbuds are undifferentiated.

The immune-histochemcial method was essentially same as Patel et al. (1989)^1^ with following modifications. After fixing in 4% paraformaldehyde, the wingbuds were washed in 1ml of 0.5% PBTx for 30 minutes at room temperature (RT). They were then washed three times in PBS with 0.01% Saponine (PBSS) for 10 minutes each at RT. The blocking was by using 500 µl of 1.5% Roche blocking solution for 30 minutes at RT with continuous mixing. Incubation with primary antibodies was also in PBSS at 4ºC with continuous mixing. After this step, the buds were washed three times in 500 µl PBSS for 20 minutes each at RT with continuous mixing. The wingbuds were incubated in Alexa Fluor anti rabbit 488 secondary at a dilution of 1:100 and 0.5 µl DAPI in 1ml PBSS for 1 hour at RT. They were mounted after washing three times with 500 µl of PBSS for 20 minutes each at RT.

*2. Generation of polyclonal antibody against Ubx of Bombyx mori*

cDNA clone corresponding to the N-terminal region of the Ubx was amplified and cloned into pET15b vector in a two step process. In the first step, primers designed to amplify (from total cDNA) the conserved regions, including the N terminal region and YPWM region and part of the homeodomain. In the second step, using the PCR product of the first step as the template, using the same forward primer, but a different reverse primer only the N-terminal region was amplified.

Primer sets used to amplify the N terminal region of *Bombyx* Ubx

Step 1:

Forward primer: 5’ GGAATTCCATATGCAGGGCGGCGGT 3’

Reverse primer: 5’ GTTGCTGTTAGCGAATGTTACAAAA 3’

Step 2:

Forward primer: 5’ GGAATTCCATATGCAGGGCGGCGGT 3’

Reverse primer: 5’ CGGGATCCGTTCGCTCCTGCTATG 3’

Expression and purification of *Bombyx* Ubx protein and generation, purification and concentrating of antibodies were as described above for *Apis* Ubx. The specificity of antibodies was confirmed by immuno-histochemistry on *Bombyx* wing buds and by Western Blot hybridization on purified proteins as well as wingbud lysates (Suppl. Fig. 1E,F). They did not cross-react to *Drosophila* Ubx (either the purified protein or the haltere disc lysate).

*3. Chromatin immune-precipitation (ChIP):*

We used a modified protocol that was used earlier for *Drosophila* wing discs^2,3^ and *Bombyx* ovarian follicles^7^.

80 wingbuds were used for as starting material for ChIP, which approximately equals to 9x10^6^ cells, were used per experiment. Nuclei from wingbuds were separated by hypotonic lysis before fixing in 1% formaldehyde (Sigma) for 12 minutes at room temperature (RT). The fixing was stopped by adding Glycine to a final concentration of 1.25 M for 5 minutes at RT. The nuclei were pelleted and washed with PBS with protease inhibitors.

The ChIP experiment was performed using a modified Invitrogen Magnify ChIP kit protocol with the reagents provided in the kit. The washed nuclei pellet was lysed using nuclei 200µl of Invitrogen Magnify lysis buffer (with Invitrogen Magnify Protease inhibitors). The tubes were left on rotator for 20 minutes at RT for complete lysis to occur and a sample was tested with DAPI stain for complete lysis which was indicated by the absence of intact nuclei. The lysate was sonicated on Diagenode Bioruptor XL for a total time of 15 minutes with 55 sec on /60 sec off cycle at high power. This setting yielded desired size (200-500bp) of chromatin fragments (Suppl. Fig. 4B). The sonicated lysate was centrifuged at 4 °C for 10 minutes at 18000g. The resultant supernatant was the chromatin to be used in the experiment and was divided equally between experiment (anti Ubx) and control (IgG) conditions while retaining 5% as input control.

Antibodies (both control and experiment) were bound to Dynabeads for six hours at 4 °C and washed with dilution buffer provided in the kit. The chromatin was diluted in the dilution buffers and allowed to bind to the antibody-Dynabead complex overnight at 4 °C. The bound chromatin was washed with low salt IP buffer for three times and high salt IP buffer twice for 5 minutes per wash at 4 °C.

After wash, the chromatin bound beads and inputs were resuspended in 50 µl de-crosslinking buffer with 2.5 µg RNAse (Roche) and incubated at 37 °C for two hours. Then the tubes were heated at 65 °C on a thermal block for 8 hours for de-crosslinking. The tubes were vortexed thoroughly to dislodge the complexes and then kept on magnetic rack to aspirate the supernatant that contained the antibody enriched chromatin. This chromatin was treated with 1 µl of Magnify Proteinase K at 55°C for two hours to get rid of the proteins. After this step the tubes were cooled on ice for 5 minutes and subjected for DNA purification. DNA purification was carried out using the magnetic DNA purification bead mixture provided in the kit. The elution was carried out twice, pooled and concentrated to completely elute the precipitated DNA.

Sequencing: The ChIP fragments were sequenced by a commercial service provider Genotypic Technology Pvt. Ltd., Bangalore using Illumina platform using standard protocols provided by the manufacturer.

Raw data is available on <http://www.ncbi.nlm.nih.gov/geo/query/acc.cgi?acc=GSE71990>.

*4. Data analysis and different bioinformatics tools*

Methods of data analysis of the sequences determined as above were essentially as described for *Apis* data. The peaks in experimental (both test and control) were identified against input DNA sequence (Suppl. Fig. 4D). The quality of *Bombyx* genome sequences (in both databases) is much lower compared to *Apis* genome sequences. Only 80% of the genome was used as effective genome size owing to very high levels of repeat sequences. Only those ChIP-seq event that provided at least 15-20 million reads was considered as a valid replicate. The peaks were filtered with following criteria to ensure a good balance between stringency and number of peaks. (A) Only those peaks were considered which had an FDR <= 15% (Suppl. Fig. 5B). (B) All the pre-immune IgG peaks were subtracted from the anti-Ubx IgG peaks. (C) The peaks those were common to both the replicates after this filtering was used for subsequent applications.

*5. Transcriptome analysis*

Wingbuds were isolated from fourth instar *Bombyx* larvae (Daizo race), the same stage that was used for ChIP experiment. Eighty each of fore- and hindwing buds were isolated and the freshly isolated buds were collected into a microcentrifuge tube kept in liquid nitrogen all the time during isolation of wingbuds. The tubes were then immediately transferred to -80ºC before isolation of RNA. The total RNA was isolated by Trizol extraction and checked for quality on a Bio-analyzer. The libraries were prepared and paired end sequencing was done on an Illumina platform. Both hindwing and forewing buds were sequenced from both ends with sequence read lengths of 100 bp. Raw data is available on <http://www.ncbi.nlm.nih.gov/geo/query/acc.cgi?acc=GSE71988>.

The reads obtained were then subjected to quality control by FastQC and the reads with low quality were trimmed. The aligner from the Cufflinks Tuxedo tools suite, TopHat 2.0.8b^8^ was used as the principle mapper to map RNA-Seq reads to the genome. Standard program Cuff links and Cuffdiff were used to assemble the transcripts and to determine differential expression. A fold change of 2 or above was used to determine the genes that considered as differentially expressed between fore- and hindwing buds.

**Nucleotide Sequences of various constructs used in this study:**

1. Full-length cDNA of *Apis* Ubx used to generate UAS-Ubx*_Apis_*

ATGAACTCGTATTTTGAGCAGACTGCGGGTGGCTTCTACGGAAGCCACCACCATCAGACAGGAGCCGCCAGTCAGCATCATGATCCAGCCACGGCAGCCGCCTATCGAAGTTTCCCCCTCGGCCTCGGTATGTCACCGTACGCGTCCACCCAACACCATCATCACACCTCCTCGTCGTTGGGCATACACCCGGGCGGTGGGACGAACACGAGGCCGCCCCAGGATTCGCCGTACGATGCGAGCGTCGCGACGGCTTGCAAGCTTTATTCGACGACGCCCGAGGCAACTGGCCACACGACATCCTCGTATTCGACCACAGCGGCCAAGGACTGTAAGCAACAGGATCAAGCATCGGCGCATCAGAACGGTTACGCCGCAGTGATGGCAGCTGCCGCCGTCAAGGACGTGTGGCAATCGGCTACCTCGGGGGCGAACAGCCAGAGCAATTCGGTGGTTCGCCCATCGGCGTGCACCCCGGAAGGGACGAGGGTTGGTAGCTACGGTGGTCTCGTAGGCGGCGATCCGGCATCGAGTCCCGGCAACAACAGTTCCTCGAGGTCCCTCACGTCGTCCTGGAACACCTGCAGTTTGAACTCGTCCGCGAGCCAACCGGTTGCCACGCAACTACATCAGCAACCCAGCAACCATACGTTCTACCCCTGGATGGCTATAGCAGGAGCGAACGGAATGCGCAGGCGCGGCCGCCAGACCTATACGCGCTACCAGACGCTCGAACTGGAGAAGGAATTCCACACGAACCACTACCTCACTAGGCGGAGGCGGATCGAGATGGCACACTCGCTCTGCCTGACGGAACGGCAGATCAAGATCTGGTTCCAGAATCGGCGGATGAAGCTGAAGAAGGAGATACAGGCGATCAAGGAGCTGAACGAACAGGAGAAGCAGGCGCAGGCGCAGAAGGCAGCGGCAGCAGCGGCCGCGGCTGCGCATCAGCAGCAAGCGGCCGGTGGGGGACCGGAGGGGGCCAACTAG

2. Full-length cDNA of *Bombyx* Ubx used to generate UAS-Ubx*_Bombyx_*

ATGAACTCTTACTTCGAGCAGGGTGGTTTTTACGGGGCCCATGGAGTGCACCAGGGCGGCGGTGGTGGAGACCAGTACCGCGGCTTCCCTCTGGGCCTCACGTATGCACAGCCACACGCTTTGCACCAGCCTCGTCCTCAGGATTCACCGTACGACGCGTCTGTCGCGGCGGCCTGCAAGCTCTATGCTGGAGAGCAGCAATATCCTAAAGCAGATTGTTCAAAGCCAGGCGGTGAGCAGCAGAATGGCTATGGTGGGAAAGAAGCCTGGGGCTCAGGTCTGGGAGCACTAGTGAGGCCGGCAGCATGCACTCCTGAAGCTCGATACAGTGAGTCGTCAAGTCCTGGTAGAGCGCTTCCGTGGGGCAACCAGTGTGCACTTCCGGGATCAGCAGCATCAGCCGCGCAGCCAGTGCACCAGCAGCCTACTAACCACACTTTCTACCCTTGGATGGCCATAGCAGGAGCGAACGGCCTCAGGAGACGAGGAAGACAAACCTACACTAGATATCAAACGCTAGAATTAGAGAAAGAGTTCCACACGAACCACTACCTTACGCGAAGGAGACGCATAGAGATGGCGCACGCGTTGTGCCTCACGGAGAGGCAAATCAAAATATGGTTCCAGAACCGAAGGATGAAGTTAAAGAAAGAGATCCAGGCTATAAAGGAGTTGAACGAGCAGGAGAAACAGGCGCAGGCGCAGAAGGCGGCAGCGGCTGCTGCGGCGGCCGCGGCTGCTGCCCAGGGACACCCCGAACATTAA

3. Full-length cDNA of *Tribolium* Ubx used to generate UAS-Ubx*_Tribolium_*

ATGAACTCTTACTTCGAGCAGAGCGGCTTCTACGGCAGCCACCACCACCAGAGCGGGTCGGTGGCGGGCCACCACCACGAGCAGTCGGCGGCGGCGGCGGCGGCCTACCGCTCCTTCCCGCTGTCGCTCGGCATGTCCCCGTACGCCTCCAGCCAGCACCACCACCACCACCTGCAGGCGCGGCCCCCGCAGGACTCGCCGTACGACGCCTCGGTGGCGGCCGCCTGCAAGCTCTACTCCTCCGAGGGCCAGCAGAACTCCAACTACTCCTCCAACTCGAAGCCGGACTGCTCCAAAGGCAACGCCGACCAGAACGGATACGCCTCGGTGGTGGCGGCGGCCGCGGTCAAGGACGTTTGGCAAAGTGCGACTTCTGGCGGTGGCGCTAATCTCACGAACAGTTTGACGGGGCCGGTCAGGCCGGCGGCATGCACGCCGGACTCCAGGGTTGGCTACGGGTCGGTCGGGCTCGTCGGCGGAGATCCGGCCTCGAGTCCGGGGGCGGCCGCAGGACGGACGGGCAACTCGCTCTCGTGGAATAACCCCTGCAGTATCAACTCGACCTCTTCGCAGCCCGTTGGCACGCAGATACACCAGCAGACCAACCACACGTTTTACCCCTGGATGGCCATTGCAGGAGCGAATGGTCTCCGAAGGCGAGGCCGACAGACGTATACCCGGTACCAGACGCTGGAGCTGGAAAAAGAGTTCCACACAAACCATTACCTGACACGGCGGCGGCGGATCGAAATGGCTCACGCACTGTGTCTTACCGAACGACAGATAAAAATCTGGTTTCAGAATCGTCGCATGAAACTCAAGAAAGAGATCCAAGCGATCAAAGAACTCAACGAGCAAGAAAAACAAGCACAGGCTCAAAAAGCGGCGGCGGCAGCTGCAGCCGTCGCCGCACAAGTGGACCCGAATTAG

6. Sequence of putative enhancer region of *vg* from *Apis* genome:

TTTCTAATCTTCTCGCGAGAAACGAGAGGCTTTCCCCGACTCTTATATAACTGGAAGTTGGACTGCGAGAATGAAAACGGCGCGGCTCTTTTTGCGGCGACGCGTCTTGGATATGTTGGAGCAGAGTCGAGGGAGGGACGGGAGGAGGTGGAGGAGGAGGAGGGAGAGCGAAATGTTTTACGGCCATGCCGTGGCTTTTTGTCGTCGGCATCGATGATTTCGGTGGGAGAACAACGAAGAAAGCACGAGTCCCGAGAGCGTGGCACCGCTATATCGGCCCCCATTAAGCTCTTATTAATTGCGAGCATCTGAGGGGCCGACCGACCTTTCTTGCGCGCTACCTGCGCGCCATTCCGCCTCCGTTCCCTCTCCGCTGCCCGCCGCAAACAAACCCGCTGCACTCCTCGGCCTTCCAACTTTGATGTCGAGTCGATCCTCCTCCAAAGAGATTTATCATGTAAATCGTGGGTAAAAAGCCCGCTCATCCTTCCCTATATCGCGACCCATCGCGTCCTCTTTTTTTCTTTTTTTCTTTTTTTTTTTTCTTCGTGTCCTCGTCACTGTCCACCGCCTTCT

7. Sequence of quadrant enhancer region of *vg* from *Drosophila* genome:

GGAGCTCCCTCCGGAGACCGGGGGCCCAAAAATAGCAACTGCAATTGAGCGGCAGGAGATACCAAAAACTTGCATAGGCTTGCATTTGCGTTGACAACATTCCAAACTCGATTCGAGAGAAAAATATCCAACAACTGGAGAGGAGTTTTGAGGATGCGGACGAGGACGAGGTCGGAGGATGTGGATGTGGATGTGCTTGTGGGGATGTGTAATGTTTGTGCTTGGCTGCCGTCGCGATTCGACAACTTTGGCCGGCACGTTGGCGAGTGTGCCATGCATGCTGATGACGATGAGAATGAGGATGAGGATGAGGATGCGGATGATGATGGTGCTGGTGCGGCTGGGATACTGAATACTGGATACGGGATGCCATGCCGCGTGCCTTTTTTTTCCCGTACCAGAAGCCAGAAGTCGTCATCATCCCATTGCCATTCACTCACTCGCTCAGCTGAGGCCGTGGAATTCCCATTAATGTGCAAACAAGCAGACTGCCAAAGATATTTCCTCTGCAGCTCCTTCAGTTAGCATTTCACTTTCAGCCAGCGGCTTCAAAAGCGAAAGCCGCACTACCTGTCCCCACCTTTCACCTTTTGGCCTAATGAAGAGAGCGTGGCGATTTATGACCCGATAACGTTCGATCGCCAGCGTTGACGCATAGTGCGGTCCTGCACAGAGAAAACTATCCTAATCCTCTATGAAGATTCTAAATCAAGTATGGAGTATTCAAACTAAAATCGACTCAACAGCAGTTTTATCTTGCTTAAACTATAGTGAGTTTCAATTAGAAACGACAAAGTACAATCGAT

**Results and Discussion**

*1. ChIP-seq in Apis mellifera*

We performed two identical ChIP experiments using anti-Ubx and pre-immune IgG as described earlier. We found that 8834 peaks were common to both the replicates after subtracting overlapping peaks observed in the negative (ChIP-seq using pre-immune IgG). Out of these, 8036 peaks had a FDR <= 1%. For further analysis, we decided to use the peaks that had a fold enrichment of 10 or greater over input, leaving us with 2350 peaks, which were used to identify putative direct targets of Ubx in *Apis* hindwing buds. Similar analysis on forewing buds yielded 927 peaks (Suppl. Fig. 5A).

We identified a total of 1396 genes that are targets of Ubx in the hindwing buds of *Apis*. Out of these, 1182 had known orthologs in *Drosophila* melanogaster (Suppl. Fig. 5A). These 1182 genes were subsequently used for all the future analysis and considered as putative targets of Ubx in hindwing buds of honeybee. To the best of our knowledge, direct target genes of Ubx in honeybee or any other hymenopterans have not been reported. Also, we had no prior knowledge about any fragment in honeybee genome that is bound by Ubx. We therefore could not use any positive controls to validate our ChIP experiment. We, therefore, used a number of parallel approaches that strengthened our observations that the Ubx-bound regions that we have identified in the current study are indeed true targets of Ubx in hindwing buds of *Apis*.

We carried out qPCR on chipped DNA samples of a third independent replicate to look for enrichment of peaks that we have identified using the first two replicates. We found that in anti-Ubx chipped samples, the peaks we identified showed a significant enrichment over input. On the contrary, we found that the fragments pulled down by pre-immune IgG showed poor or no enrichment over input (Suppl. Fig. 5E). This showed that the peaks that we have identified using bioinformatics approach in the two replicates are true peaks or regions bound by Ubx, which are also pulled down in an independent ChIP replicate. We noticed that most pulled down sequences were within 2kb of a coding gene.

*2. ChIP-seq in Bombyx mori*

At 15% cut-off and subsequent filtering as described above, we identified 340 peaks for forewing buds and 1128 peaks for hindwing buds as potential Ubx binding sites in the *Bombyx* genome (Suppl. Fig. 5B). These corresponded to 245 genes and 871 genes, respectively, which were considered as putative targets of Ubx in *Bombyx* (Suppl. Fig. 5B). Only 28 peaks in the hindwing ChIP-seq data were left unassigned.

**References**

1 Patel, N.H., Martin-Blanco, E., Coleman, K.G., Poole, S.J., Ellis, M.C., Kornberg, T. B. and Goodman, C.S. Expression of Engrailed proteins in arthropods, annelids, and chordates. *Cell* **58**, 955–968. (1989).

2 Agrawal, P., Habib, F., Yelagandula, R. and Shashidhara, L.S. Genome-level identification of targets of Hox protein Ultrabithorax in *Drosophila*: novel mechanisms for target selection. *Sci. Rep.* 1:1–10. (2011).

3 Agrawal, P. and Shashidhara, L.S. 2014. ChIP for Hox Proteins from *Drosophila* Imaginal Discs. In Hox Genes. Springer New York. 1196:241–253.

4 Zhang, et al. Model-based Analysis of ChIP-Seq (MACS). *Genome Biol.* **9**, R137. (2008).

5 Hojyo, T. and Fujiwara, H. Reciprocal transplantation of wing discs between a wing deficient mutant (fl) and wild type of the silkworm, *Bombyx mori.* *Dev Growth Differ.* 39, 599-606. (1997).

6 Kango-Singh, M., Singh, A. and Gopinathan, K.P. The wings of *Bombyx* *mori* develop from larval discs exhibiting an early differentiated state: a preliminary report. *J. Biosci.* 26:167–177. (2001).

7 Papantonis, A. and Lecanidou, R. A modified chromatin-immunoprecipitation protocol for silkmoth ovarian follicular cells reveals C/EBP and GATA binding modes on an early chorion gene promoter. *Molecular Biology Reports* **36**: 733-736. (2009).

8 Trapnell, C. et al. Differential gene and transcript expression analysis of RNA-Seq experiments with TopHat and Cufflinks. *Nature Protocols* **7**, 562–78. (2012).

9 Choo, S. W., White, R. & Russell, S. Genome-Wide Analysis of the Binding of the Hox Protein Ultrabithorax and the Hox Cofactor Homothorax in Drosophila. *PLoS ONE* **6**, e14778, doi:10.1371/journal.pone.0014778 (2011).

**Supplement Table 1**

Putative Targets of *Drosophila* Ubx^9^ that are common to those in both *Apis* and *Bombyx*.

| **Flybase ID** | **GeneID** | **Symbol** | **Gene name** |
| --- | --- | --- | --- |
| FBgn0000179 | CG3578 | bi | bifid |
| FBgn0000542 | CG2904 | ec | echinus |
| FBgn0000546 | CG1765 | EcR | Ecdysone receptor |
| FBgn0000568 | CG8127 | Eip75B | Ecdysone-induced protein 75B |
| FBgn0000577 | CG9015 | en | engrailed |
| FBgn0002643 | CG8118 | mam | mastermind |
| FBgn0003079 | CG2845 | phl | pole hole |
| FBgn0003415 | CG9936 | skd | skuld |
| FBgn0003975 | CG3830 | vg | vestigial |
| FBgn0004644 | CG4637 | hh | hedgehog |
| FBgn0004893 | CG10021 | bowl | brother of odd with entrails limited |
| FBgn0004907 | CG17870 | 14-3-3zeta | 14-3-3zeta |
| FBgn0005771 | CG4491 | noc | no ocelli |
| FBgn0010575 | CG5580 | sbb | scribbler |
| FBgn0014037 | CG32217 | Su(Tpl) | Su(Tpl) |
| FBgn0016977 | CG18497 | spen | split ends |
| FBgn0020257 | CG9952 | ppa | partner of paired |
| FBgn0029114 | CG6890 | Tollo | Tollo |
| FBgn0030065 | CG12075 | CG12075 | - |
| FBgn0032120 | CG33298 | CG33298 | - |
| FBgn0035101 | CG1212 | p130CAS | p130CAS |
| FBgn0037120 | CG11247 | CG11247 | - |
| FBgn0037305 | CG12173 | CG12173 | - |
| FBgn0039907 | CG2041 | lgs | legless |
| FBgn0041094 | CG7590 | scyl | scylla |
| FBgn0086899 | CG34412 | tlk | Tousled-like kinase |
| FBgn0260635 | CG12284 | th | thread |
| FBgn0261383 | CG3125 | IntS6 | Integrator 6 |
| FBgn0262127 | CG33967 | kibra | kibra ortholog |
| FBgn0262656 | CG10798 | dm | diminutive |
| FBgn0262719 | CG43163 | CG43163 | - |
| FBgn0262735 | CG1691 | Imp | IGF-II mRNA-binding protein |
| FBgn0263396 | CG16901 | sqd | squid |

**Figures and Figure Legends**

**Suppl. Figure 1**

**
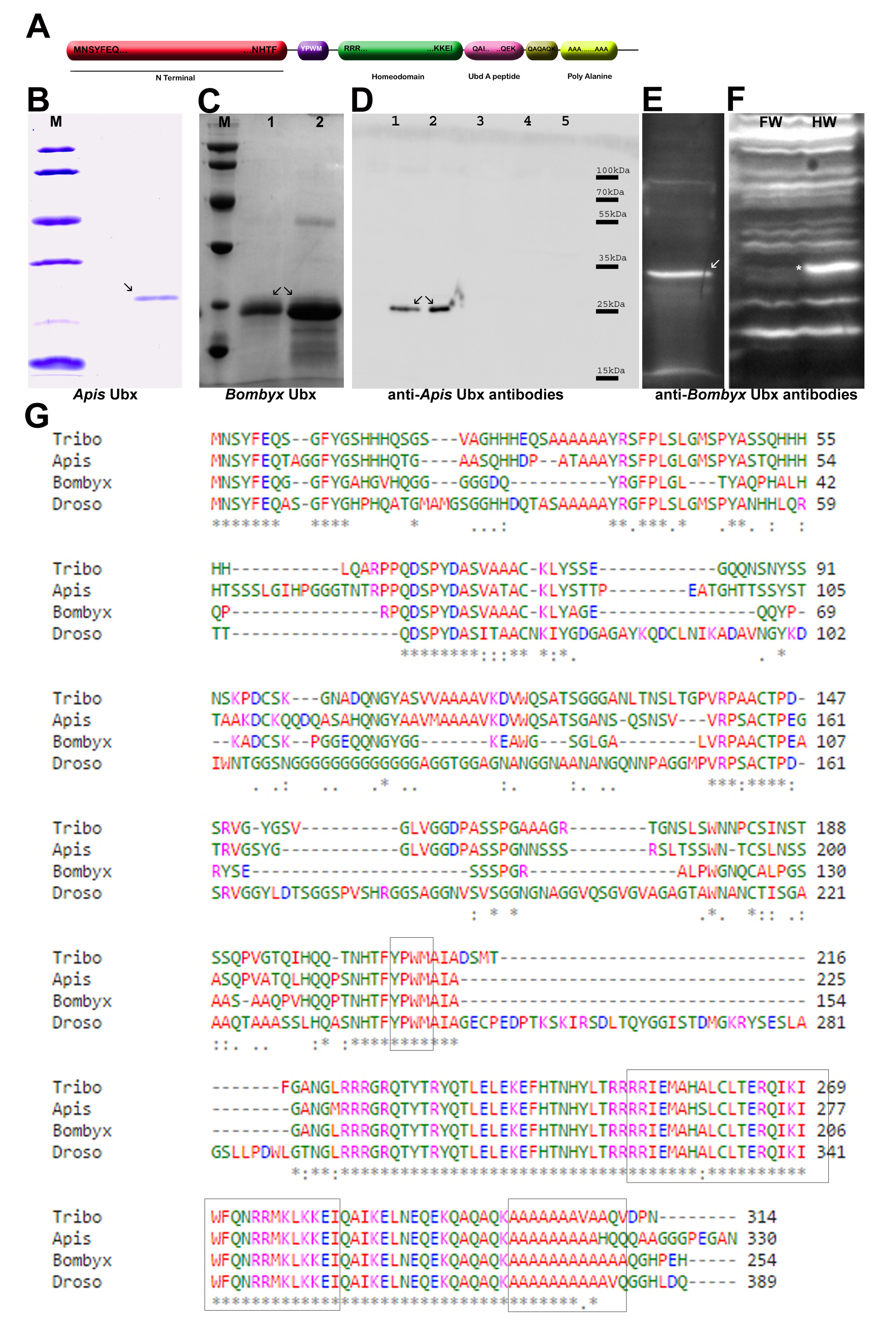
**

**Suppl. Figure 1:** (A) Schematic drawing of various functional domains in *Drosophila* Ubx. This domain structure if very similar to both *Apis* and *Bombyx* Ubx. The N-terminal region is unique to Ubx, while the homeodomain is conserved with other Hox and some non-Hox TFs. (B-C) Expression and purification of N-terminus fragment of *Apis* (B) and *Bombyx* (C) Ubx. The Ubx band of expected size is shown using arrows. In C, lane 1 and lane 2 correspond to two different purification batches. Purified protein of lane 1 was used for generating antibodies. (D-F) Western blot Hybridization using antibodies raised against *Apis* Ubx (D) or *Bombyx* Ubx (E,F). In D, lane 1 and 2: *Apis* embryonic lysates, lanes 3-4: *Drosophila* embryonic lysates and lane 5: protein molecular weight markers. In E, *Bombyx* larval lysate. In F, lysates of *Bombyx* forewing (FW) and hindwing (HW) buds.

**Suppl. Figure 2**

**
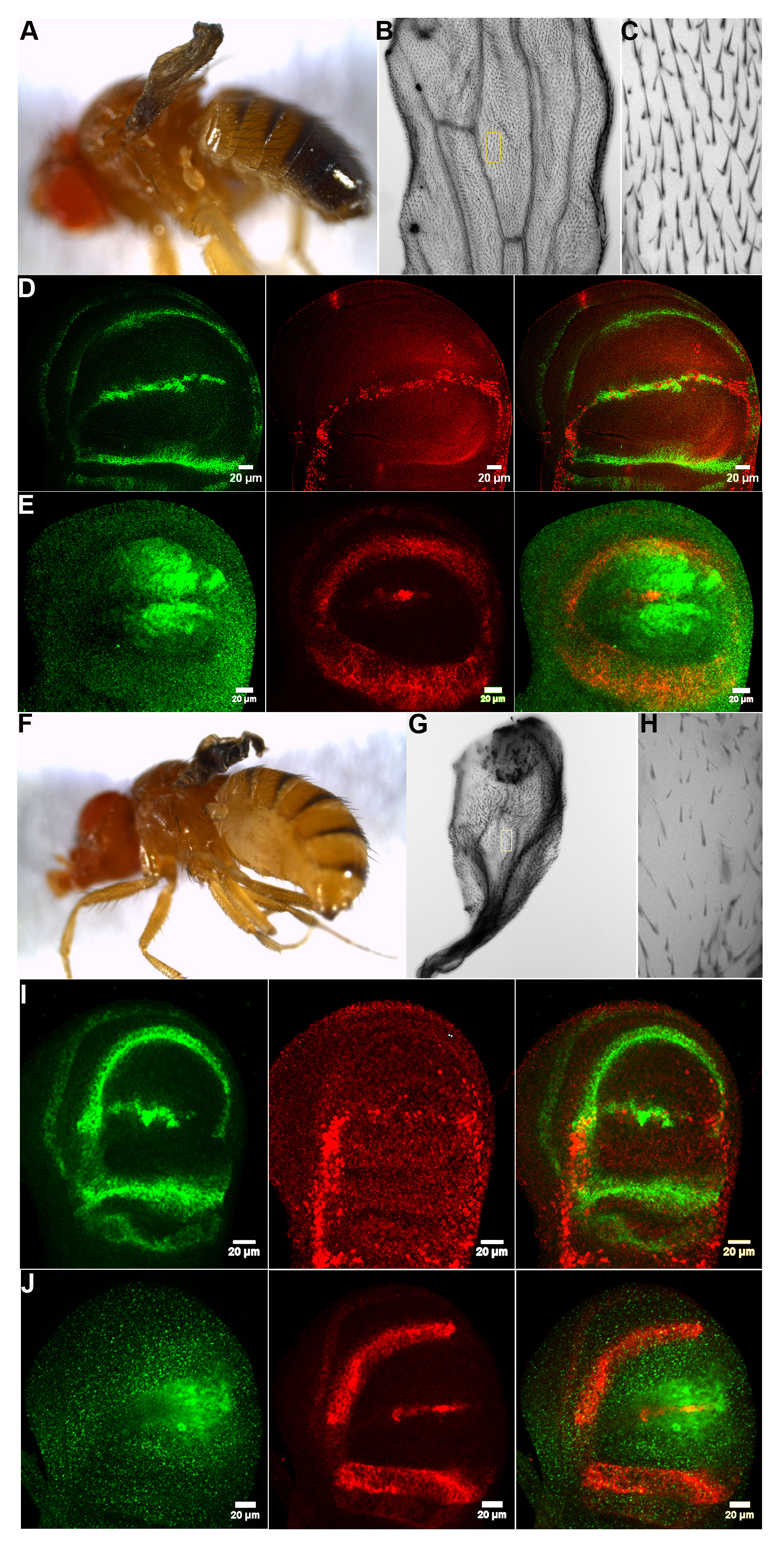
**

**Suppl. Figure 2:** Wing to haltere transformations in *Drosophila* caused by the over-expression of Ubx*_Bombyx_* or Ubx*_Tribolium_*. (A-C) *vg*-GAL4/UAS-Ubx*_Bombyx_* adult fly with reduced and deformed wing. The separated wing is shown at higher magnifications (B,C). Unlike with Ubx*_Apis_* (Fig. 3), no significant transformation at the trichome-level was observed. (D) *vg*-GAL4/UAS-Ubx*_Bombyx_* larval wing disc stained for Wg (green) and Ubx*_Bombyx_* (red). Note repression of Wg by Ubx*_Bombyx_* is only in the posterior compartment, an indication of homeotic transformation. (E) *vg*-GAL4/UAS-Ubx*_Bombyx_* larval wing disc stained for *vg* quadrant-lacZ (green) and Wg (red). Note non cell-autonomous repression of *vg* quadrant-lacZ. Repression of Wg in the posterior compartment is more pronounced in E than in D. (F-H) *vg*-GAL4/UAS-Ubx*_Tribolium_* adult fly with reduced and deformed wing. The separated wing is shown at higher magnifications (G,H). Trichomes are much shorter, although they are sparsely arranged as in wing blades. (I) *vg*-GAL4/UAS- Ubx*_Tribolium_* larval wing disc stained for Wg (green) and Ubx*_Tribolium_* (red). Note repression of Wg by Ubx*_Tribolium_* is only in the posterior compartment, an indication of homeotic transformation. (J) *vg*-GAL4/UAS-Ubx*_Tribolium_* larval wing disc stained for *vg* quadrant-lacZ (green) and Wg (red). Note non cell-autonomous repression of *vg* quadrant-lacZ. In all imaginal discs, posterior is to the right and ventral to the top.

**Suppl. Figure 3**

**
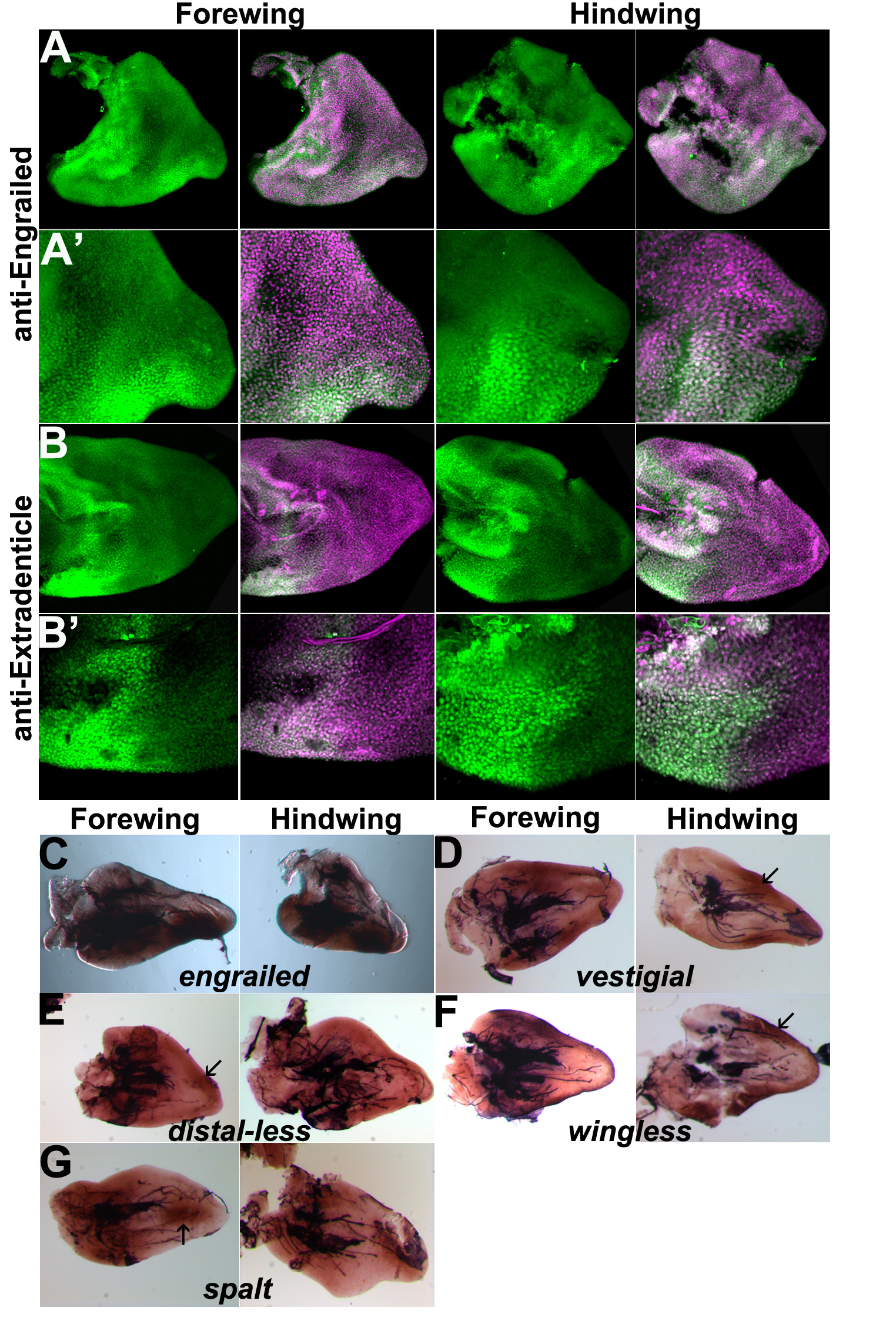
**

**Suppl. Figure 3:** Many wing-patterning genes are expressed in *Apis* wing buds in the same pattern as in *Drosophila* wing discs. (A,B) Forewing and hindwing buds of *Apis* stained for En (A) and Extradenticle (B) using antibodies against their *Drosophila* homologues. Their expression patterns in *Apis* are similar to the patterns seen in *Drosophila* wing discs. (C-G) Forewing and hindwing buds of *Apis* stained for transcripts of *en* (C), *vg* (D), *Distal-less* (E), *wg* (F) and *spalt* (G) by RNA in situ. Note while the signal is weak and there is much interference by large trachea, patterns (arrows) are similar to what one would see in *Drosophila* wing discs. RNA in situ for *en* and *spalt* confirmed the expression patterns detected by immunohistochemistry using antibodies against their counterparts in *Drosophila*. *en* is expressed only in the posterior compartment. Unlike in Lepidoptera, where two posterior compartments have been predicted, *Apis* wing buds show only one posterior compartment. In all images, proximal end is shown towards the left and the distal end towards the right. Posterior end is shown towards the bottom.

**Suppl. Figure 4**

**
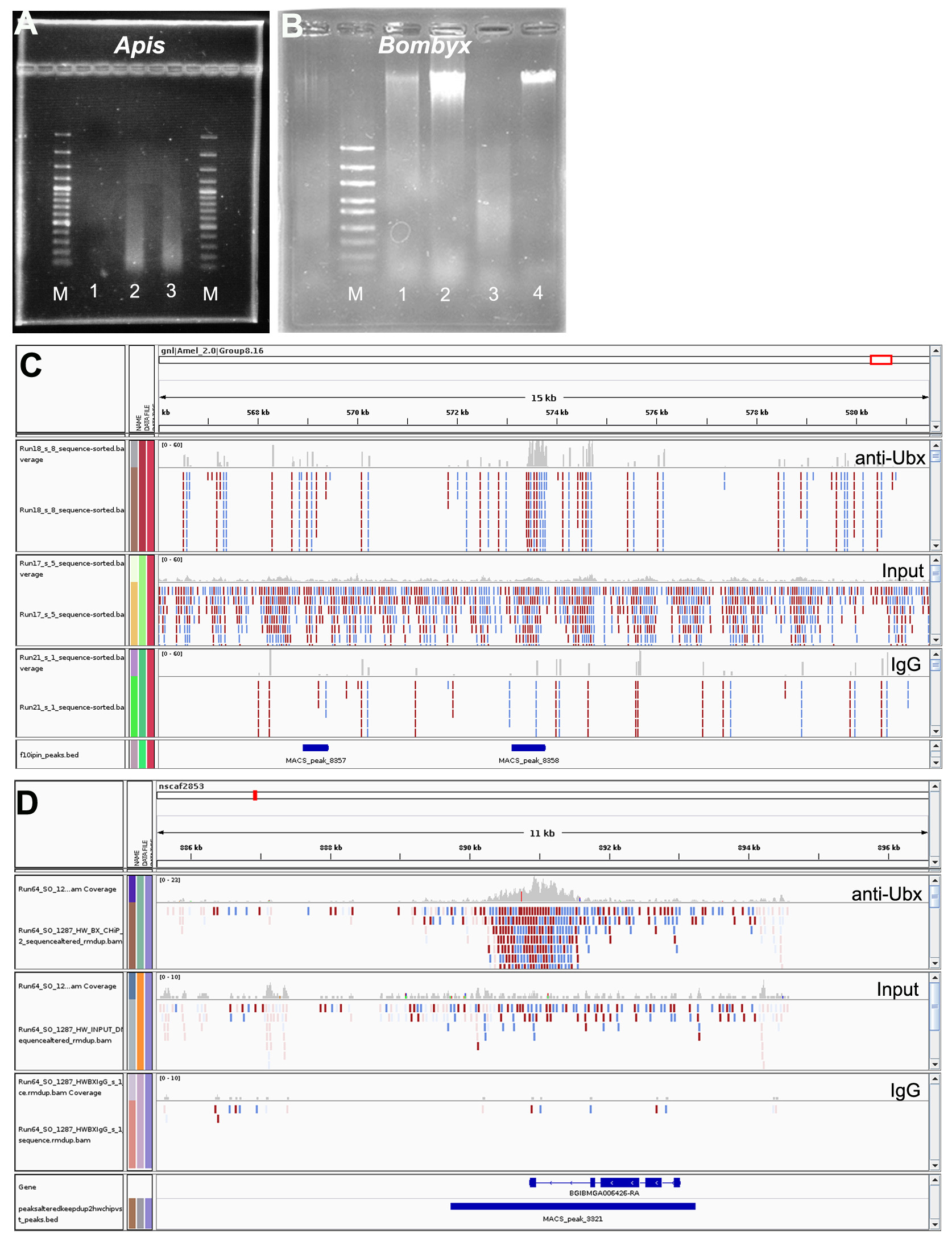
**

**Suppl. Figure 4:** ChIP standardization and data analysis. (A) Standardization of sonication time to shear the chromatin from *Apis* wing buds to 200-500bp long fragments. Lane 1: blank, Lanes 2,3: 10 and 20 min of sonication. Subsequent experiments were with 10min sonication. (B) Standardization of sonication time to shear the chromatin from *Bombyx* wing buds to 200-500bp long fragments. Lanes 1,2,3: 5, 10, 15 min of sonication. Subsequent experiments were with 15min sonication. Lane 4: genomic DNA without any sonication. (C,D) Representative images showing the filtering steps used for identifying peaks from raw sequence files using MACS. Peaks of sequences of fragments pulled down using specific antibodies were compared to peaks obtained for input control and pre-immune IgG control. Only those peaks, which were specific to the experimental ChIP were considered as positive peaks.

**Suppl. Figure 5**

**
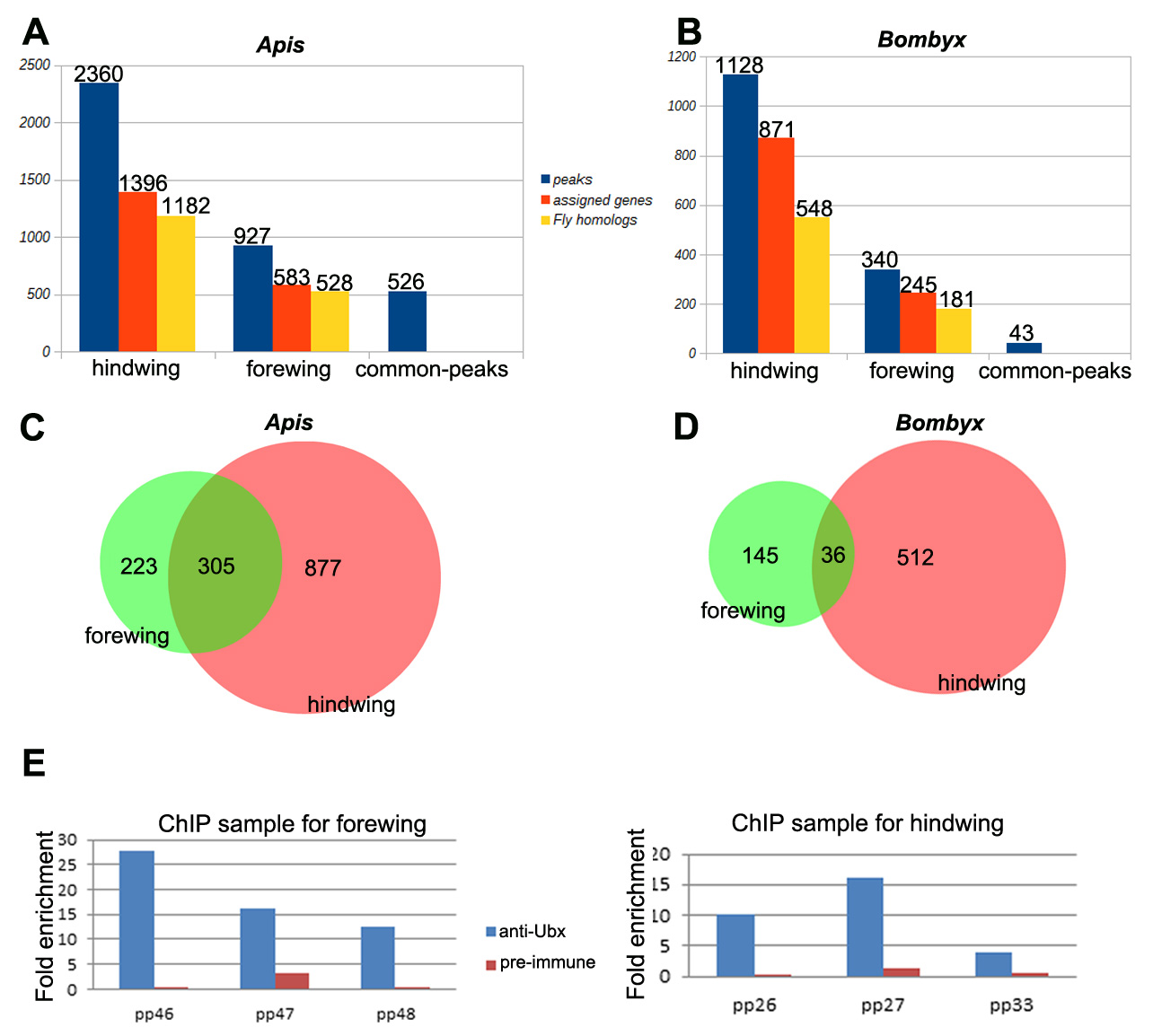
**

**Suppl. Figure 5:** Preliminary analysis of ChIP-seq data. (A, B) Peaks to genes conversion for *Apis* hindwing and forewing (A) and *Bombyx* hindwing and forewing (B). While peaks/corresponding genes common to forewing and hindwing are substantial for *Apis*, they were much fewer for *Bombyx*. (C,D) Venn diagrams showing comparison of targets of Ubx between forewing and hindwing buds of *Apis* (C) and *Bombyx* (D). (E) Validation of few positives of ChIP-seq in *Apis* by ChIP-qPCR. Note all genes show considerable enrichment in DNA fragments in ChIP using anti-Ubx*_Apis_* antibodies compared to pre-immune control.

**Suppl. Figure 6**

**
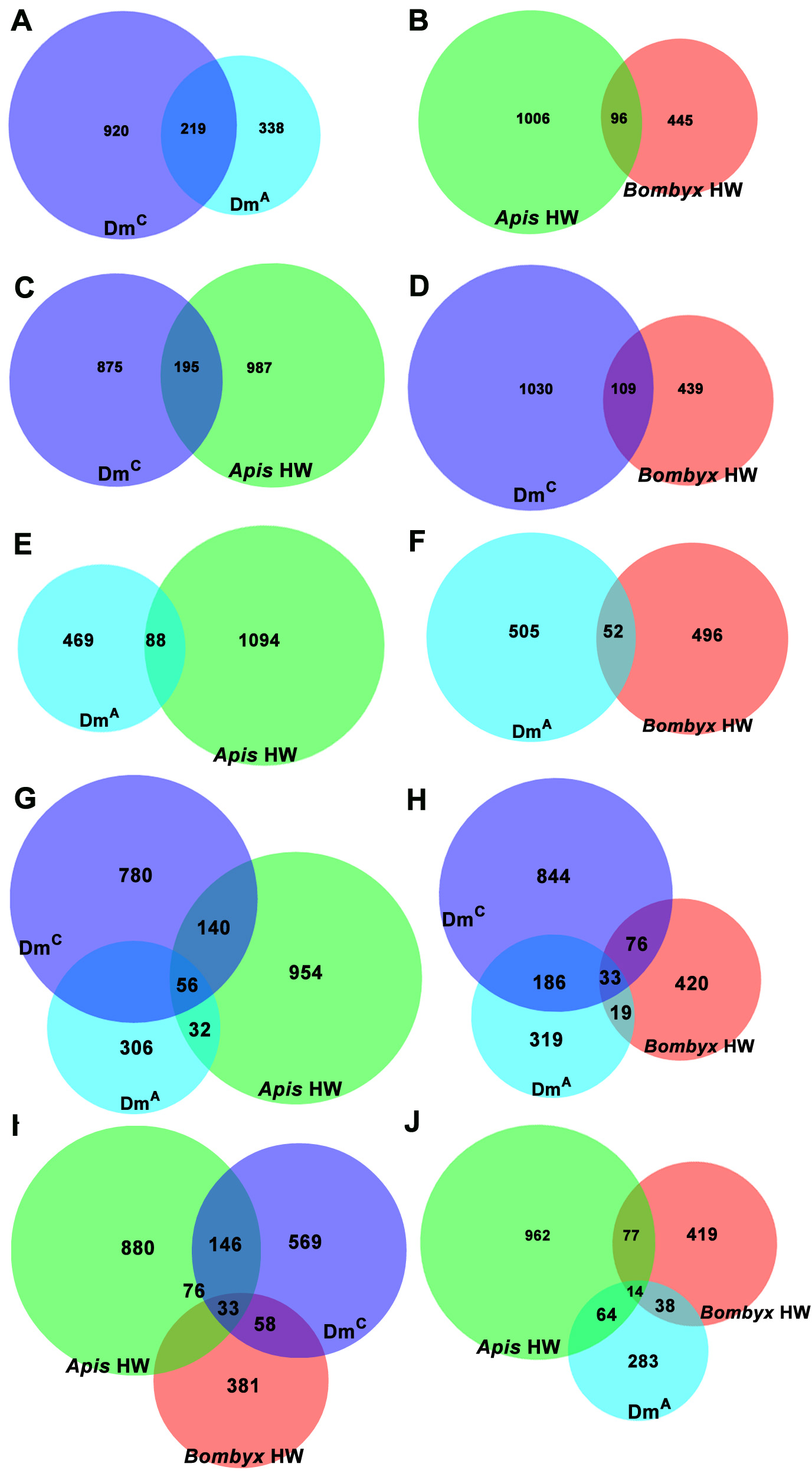
**

**Suppl. Figure 6:** Venn diagrams of comparison of targets of Ubx in *Drosophila*, *Apis* and *Bombyx*. (A) Comparison targets of *Drosophila* Ubx between two studies, Choo et al. (2011; Dm^C^) and Agrawal et al. (2011; Dm^A^). (B) Comparison targets of Ubx in *Apis* and *Bombyx* hindwing buds. (C,D) Comparison between targets of Ubx in *Drosophila* (Dm^C^) and those identified in this report for *Apis* (C) and *Bombyx* (D) hindwing buds. (E,F) Comparison between targets of Ubx in *Drosophila* (Dm^A^) and those and in *Apis* (E) and *Bombyx* (F) hindwing buds. (G,H) Three-way comparisons of targets of Ubx from two studies in *Drosophila* (Dm^C^ and Dm^A^) and in *Apis* (G) and *Bombyx* (H) hindwing buds. (I,H) Three-way comparisons of targets of Ubx in *Apis* and *Bombyx* hindwing buds and those in *Drosophila*, either Dm^C^ (I) or Dm^A^ (H). Numbers of species-specific or common targets are shown on the diagrams. 15-20% of targets of Ubx in *Drosophila* wing disc are common to those in *Apis* and *Bombyx* hindwing buds. Only those targets of Ubx in *Apis* and *Bombyx* hindwing buds, for which orthologues exist in *Drosophila* genome are considered for all comparisons including between *Apis* and *Bombyx*.

**Suppl. Figure 7**

**
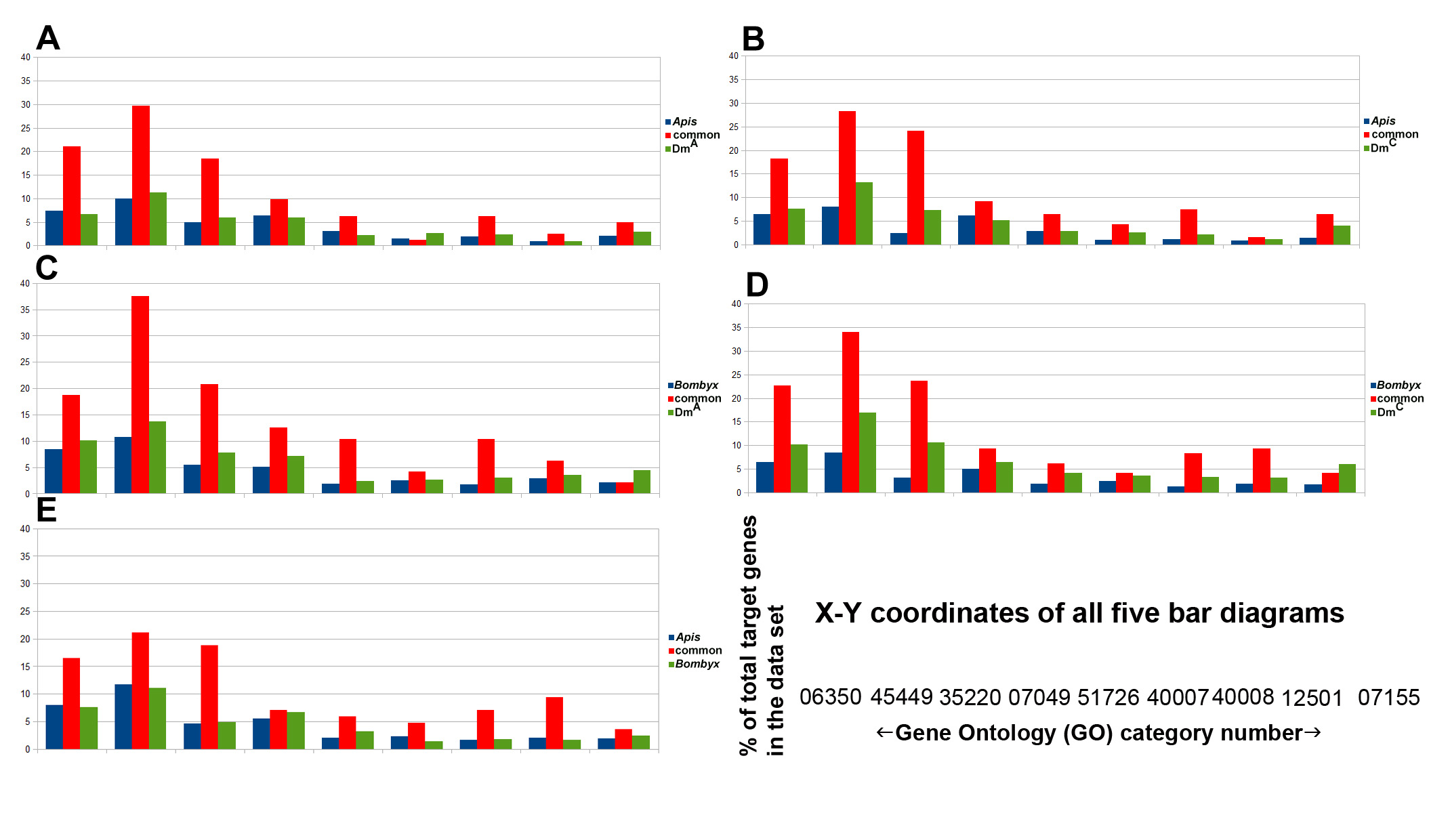
**

**Suppl. Figure 7:** Bar diagrams showing proportion of genes for a given ontological group amongst the targets that are either specific to a given species or common between two species (as indicated on the images). Dm^C^ corresponds to targets identified by Choo et al. (2011)^9^. Dm^A^ corresponds to targets identified by Agrawal et al. (2011)^2^. (A-B) Comparison between *Apis* and *Drosophila*. (C-D) Comparison between *Bombyx* and *Drosophila*. (E) Comparison between *Apis* and *Bombyx*. Gene ontological groups analysed are as follows: GO-06350- transcription; GO-45449- regulation of transcription; GO-35220- wing development; GO-07049- cell cycle; GO-51726 – regulation of cell cycle; GO-40007- growth; GO-40008- regulation of growth; GO-12501- programmed cell death; GO-07155- cell adhesion. Note, proportion of genes implicated in all the functional groups shown here are much higher amongst the genes that are common between any two species compared to species specific targets. Gene ontology is based on the information available for *Drosophila* genes.

**Suppl. Figure 8**

**
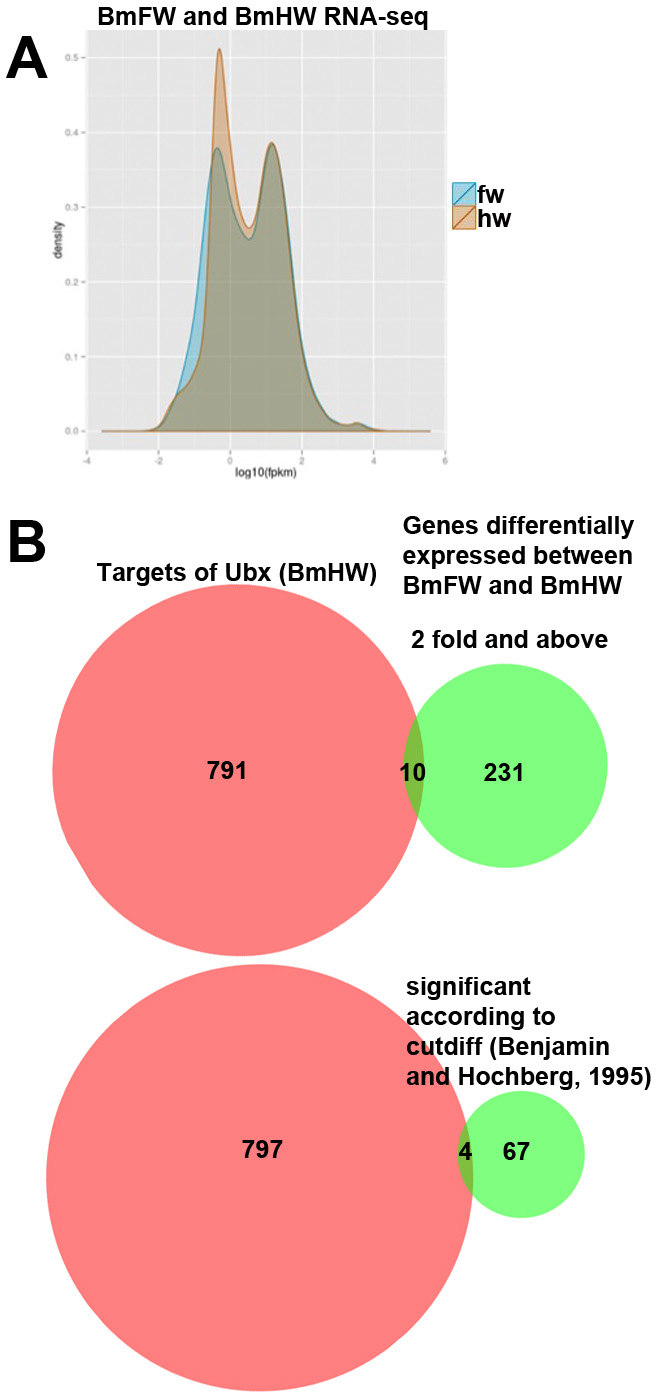
**

**Suppl. Figure 8**: Transcriptome analysis of *Bombyx* forewing and hindwing buds suggests near-identical expression profile of genes between the two wing buds. (A) Graphical representation of total transcripts that are different between forewing (fw) and hindwing (hw) buds. Number of transcripts that show different levels between the two wing buds are marginal. (B) Venn diagrams showing the overlap between direct targets of Ubx identified in this study by ChIP-seq and genes that are differentially expressed between forewing and hindwing buds as determined in this study by RNA-seq. Two different statistical methods were used by the RNA-seq analysis program to identify transcripts (and corresponding genes) whose expression levels are significantly different between the two wing buds.

**Suppl. Figure 9**

**
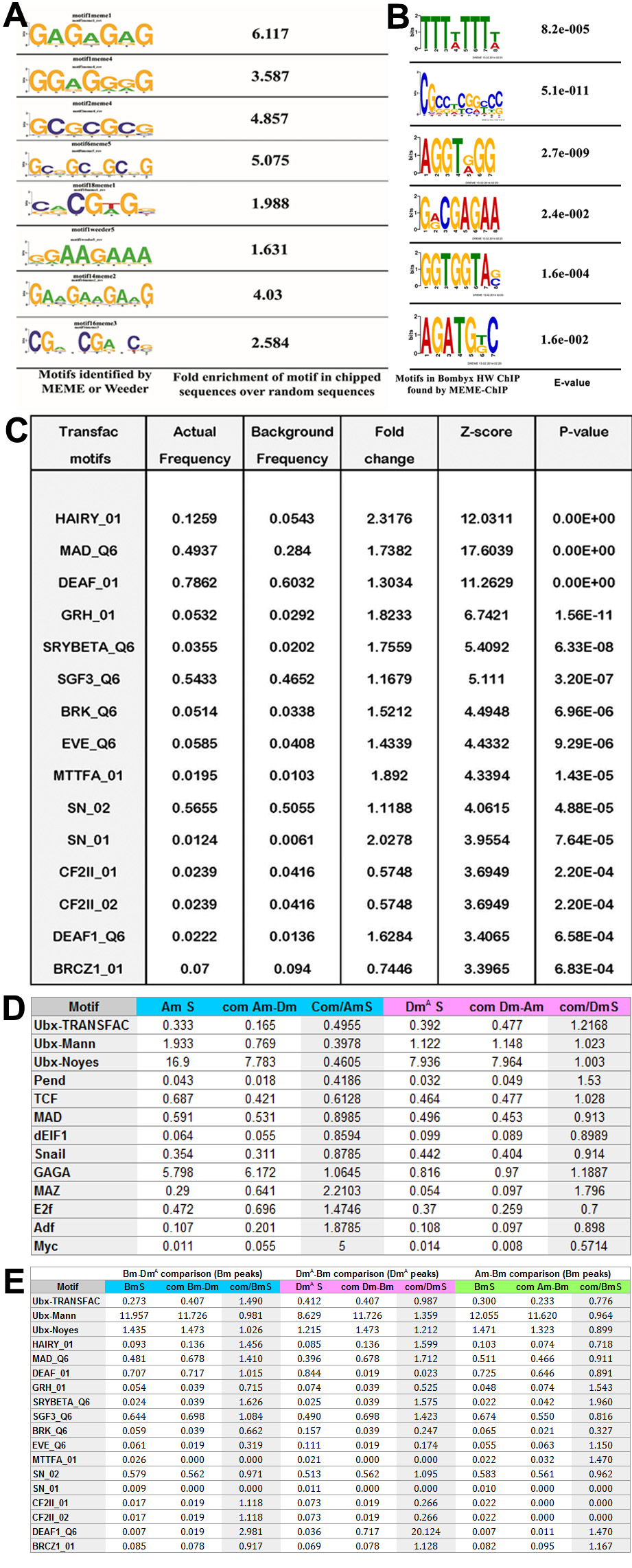
**

**Suppl. Figure 9:** Analyses of putative enhancer sequences of targets of Ubx in *Apis* and *Bombyx* at the levels of motifs recognized and bound by various other TFs, which may function in concert with Ubx. (A) Frequently occurred motifs in the sequences pulled down by anti-Ubx*_Bombyx_* from *Bombyx* hindwing buds identified by MEME, a de novo method. (B) Enrichment of binding sites for various TFs in the sequences pulled down by anti-Ubx*_Bombyx_* from *Bombyx* hindwing buds using TRANSFAC. Motifs are based on information available for TFs available in *Drosophila*. Both the methods show enrichment of similar motifs. (C) TRANSFAC analysis done as in B is shown in tabular form. Similar enrichment for binding sites for GAF, Adf-1, E2F etc. is reported for *Drosophila*^2^. (D,E) Frequency of binding sites for various TFs identified using TRANSFAC as above in the non-coding sequences of targets of Ubx that are *Apis*-specific or *Drosophila*-specific or common to both (D) or *Bombyx*-specific or *Drosophila*-specific or common to both (E). Similar comparison between *Bombyx* and *Apis* is shown in E. All the three datasets for both the insects showed similar frequencies of occurrence of a given TF in the regulatory regions of the targets of Ubx.

**Suppl. Figure 10**

**
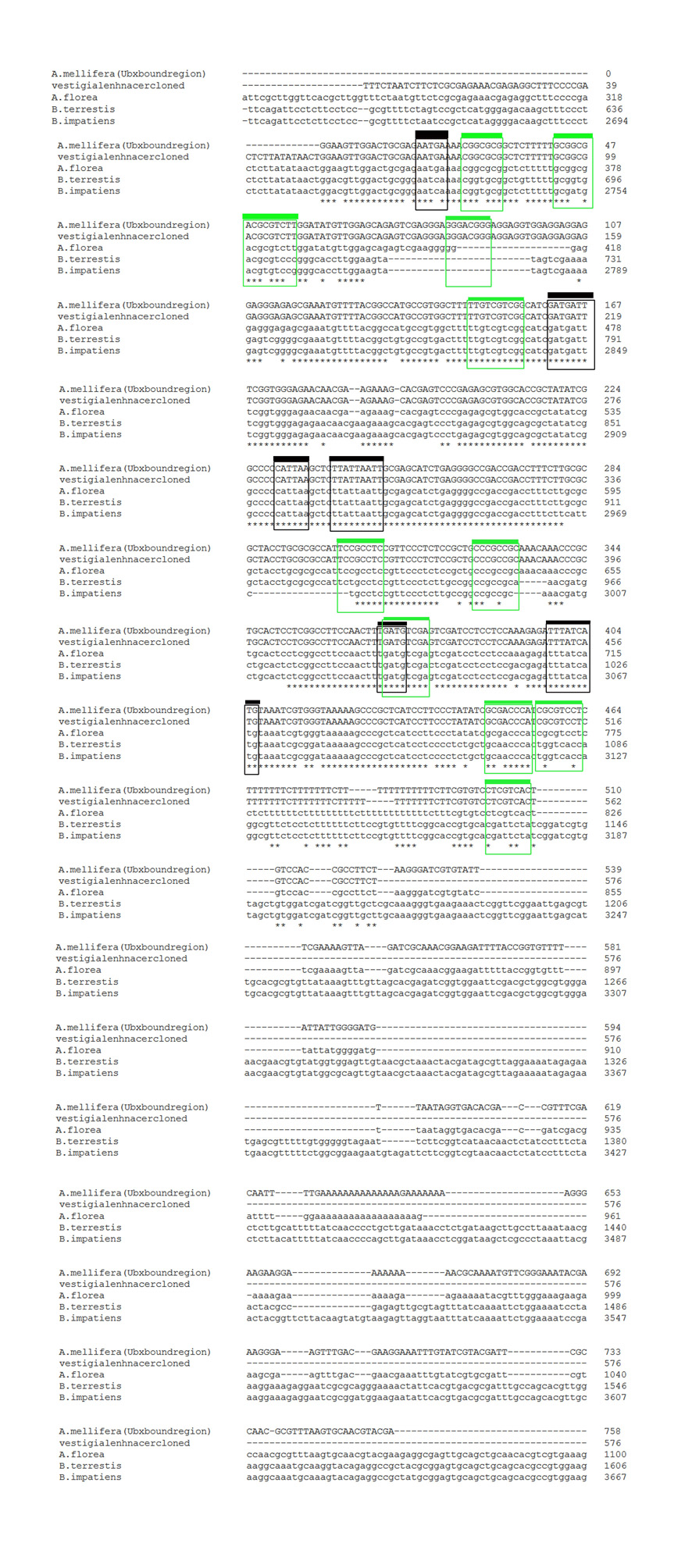
**

**Suppl. Figure 10:** Comparison of sequences of Ubx-bound region in the 4th intron of *vg* gene of different hymenopterans (*A. florea*, *B. terrestis* and *B. impatiens*). A. mellifera_Ubx bound region is the fragment bound by Ubx (as detected by our ChIP-seq study). Vgenhancercloned is the sequence of *vg* enhancer that was cloned and tested experimentally in transgenic flies. Note very high degree of homology of these non-coding sequences amongst the hymenopteran insects. Ubx-binding sites are shown in black and MAD-binding sites in green.

**Suppl. Figure 11**

**
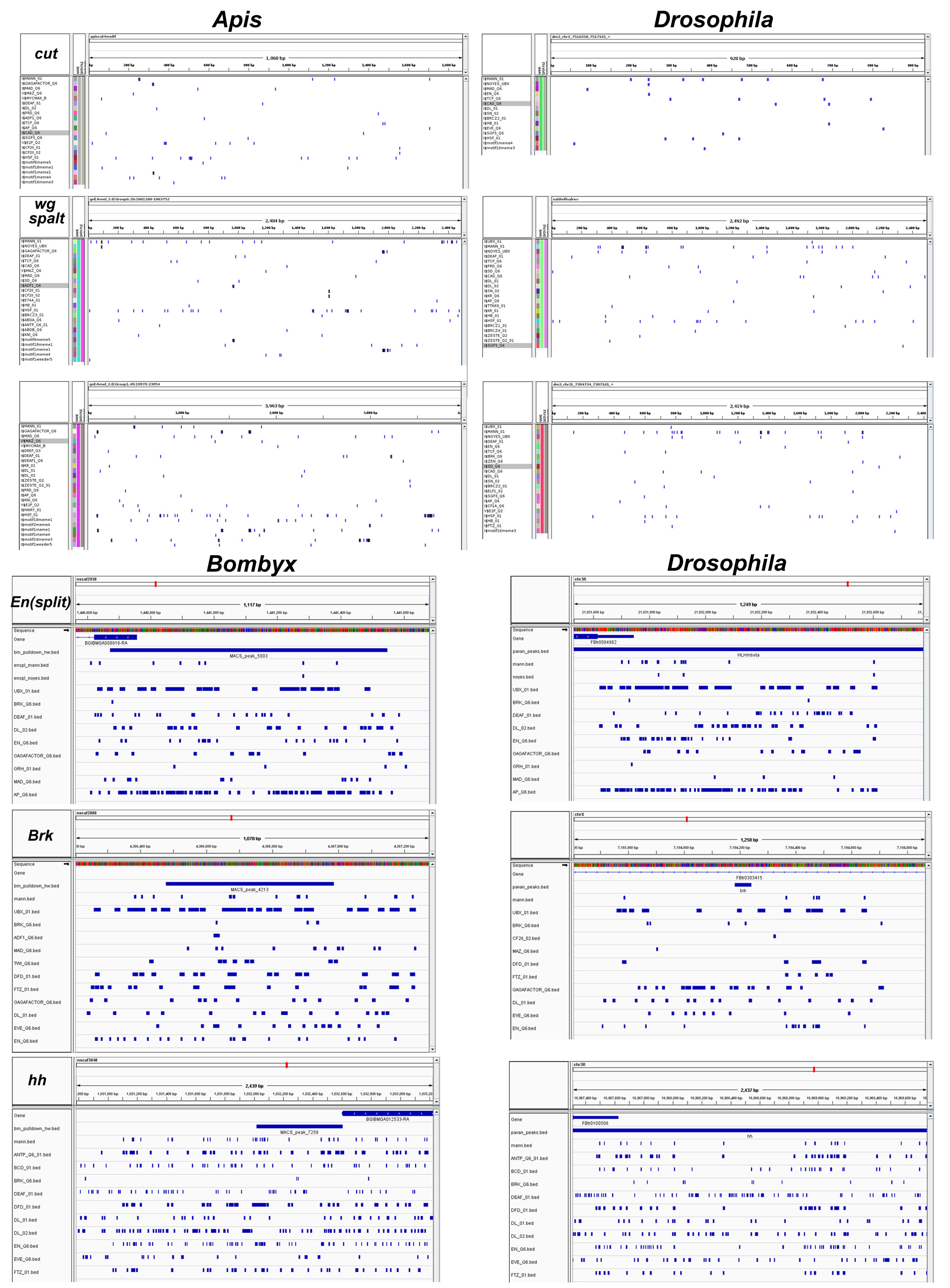
**

**Suppl. Figure 11:** TRANSFAC analyses of regulatory sequences of various targets of Ubx and the comparison of the same between *Drosophila* and *Apis* or *Drosophila* and *Bombyx*. Note a similar array of TFs bind around Ubx binding sites all the species. Few differences observed could be the reason for their differential regulation in *Drosophila* and not in other two species.
